# Rare coding variants in *CHRNB2* reduce the likelihood of smoking

**DOI:** 10.1038/s41588-023-01417-8

**Published:** 2023-06-12

**Authors:** Veera M. Rajagopal, Kyoko Watanabe, Joelle Mbatchou, Ariane Ayer, Peter Quon, Deepika Sharma, Michael D. Kessler, Kavita Praveen, Sahar Gelfman, Neelroop Parikshak, Jacqueline M. Otto, Suying Bao, Shek Man Chim, Elias Pavlopoulos, Andreja Avbersek, Manav Kapoor, Esteban Chen, Marcus B. Jones, Michelle Leblanc, Jonathan Emberson, Rory Collins, Jason Torres, Pablo Kuri Morales, Roberto Tapia-Conyer, Jesus Alegre, Jaime Berumen, GHS-REGN DiscovEHR collaboration, Lance J. Adams, Jackie Blank, Dale Bodian, Derek Boris, Adam Buchanan, David J. Carey, Ryan D. Colonie, F. Daniel Davis, Dustin N. Hartzel, Melissa Kelly, H. Lester Kirchner, Joseph B. Leader, David H. Ledbetter, J. Neil Manus, Christa L. Martin, Raghu P. Metpally, Michelle Meyer, Tooraj Mirshahi, Matthew Oetjens, Thomas Nate Person, Christopher Still, Natasha Strande, Amy Sturm, Jen Wagner, Marc Williams, Regeneron Genetics Center, Aris Economides, Andrew Deubler, Katia Karalis, Luca A. Lotta, John D. Overton, Jeffrey G. Reid, Katherine Siminovitch, Lyndon J. Mitnaul, Alan Shuldiner, Adolfo Ferrando, Christina Beechert, Caitlin Forsythe, Erin D. Brian, Zhenhua Gu, Michael Lattari, Alexander Lopez, Maria Sotiropoulos, Manasi Pradhan, Kia Manoochehri, Ricardo Schiavo, Raymond Reynoso, Kristy Guevara, Laura M. Cremona, Chenggu Wang, Hang Du, Sarah E. Wolf, Amelia Averitt, Nilanjana Banerjee, Dadong Li, Sameer Malhotra, Justin Mower, Jay Sundaram, Aaron Zhang, Sean Yu, Mudasar Sarwar, Jeffrey C. Staples, Xiaodong Bai, Lance Zhang, Sean O’Keeffe, Andrew Bunyea, Lukas Habegger, Boris Boutkov, Gisu Eom, Alicia Hawes, Olga Krasheninina, Rouel Lanche, Adam J. Mansfield, Evan Edelstein, Sujit Gokhale, Alexander Gorovits, Evan K. Maxwell, Ju Guan, George Mitra, Janice Clauer, Mona Nafde, Vrushali Mahajan, Razvan Panea, Koteswararao Makkena, Krishna PawanPunuru, Benjamin Sultan, Sanjay Sreeram, Tommy Polanco, Ayesha Rasool, William J. Salerno, Kathie Sun, Joshua Backman, Anthony Marcketta, Bin Ye, Lauren Gurski, Nan Lin, Jan Revez, Yuxin Zou, Jack Kosmicki, Jonathan Ross, Andrey Ziyatdinov, Eli Stahl, Akropravo Ghosh, Lei Chen, Rujin Wang, Adam Locke, Carlo Sidore, Arden Moscati, Lee Dobbyn, Blair Zhang, Christopher Gillies, Michael Kessler, Maria Suciu, Timothy Thornton, Priyanka Nakka, Sheila Gaynor, Tyler Joseph, Benjamin Geraghty, Anita Pandit, Joseph Herman, Sam Choi, Peter VandeHaar, Liron Ganel, Kuan-Han Wu, Aditeya Pandey, Kathy Burch, Adrian Campos, Scott Vrieze, Sailaja Vedantam, Charles Paulding, Amy Damask, Aysegul Guvenek, George Hindy, Jan Freudenberg, Jonas Bovijn, Mary Haas, Moeen Riaz, Niek Verweij, Olukayode Sosina, Parsa Akbari, Tanima De, Gannie Tzoneva, Jin He, Silvia Alvarez, Kayode Sosina, Jacqueline Otto, Anna Alkelai, Vijay Kumar, Peter Dombos, Amit Joshi, Sarah Graham, Luanluan Sun, Antoine Baldassari, Jessie Brown, Cristen J. Willer, Arthur Gilly, Hossein Khiabanian, Brian Hobbs, Billy Palmer, Juan Rodriguez-Flores, Jaimee Hernandez, Michelle G. LeBlanc, Jason Mighty, Nirupama Nishtala, Nadia Rana, Jennifer Rico-Varela, Randi Schwartz, Thomas Coleman, Alison Fenney, Jody Hankins, Ruan Cox, Samuel Hart, Alan R. Shuldiner, Suganthi Balasubramanian, Gonçalo R. Abecasis, Hyun M. Kang, Jonathan Marchini, Eli A. Stahl, Eric Jorgenson, Robert Sanchez, Wolfgang Liedtke, Matthew Anderson, Michael Cantor, David Lederer, Aris Baras, Giovanni Coppola

**Affiliations:** 1 Regeneron Genetics Center, Tarrytown, NY USA; 2 Regeneron Pharmaceuticals, Inc., Tarrytown, NY USA; 3 Clinical Trial Service Unit and Epidemiological Studies Unit, Nuffield Department of Population Health, University of Oxford, Oxford, UK; 4 MRC Population Health Research Unit, Nuffield Department of Population Health, University of Oxford, Oxford, UK; 5 Experimental Research Unit from the Faculty of Medicine (UIME), National Autonomous University of Mexico (UNAM), Mexico, Mexico; 6 Instituto Tecnológico y de Estudios Superiores de Monterrey, Monterrey, Mexico; 7 Geisinger, Danville, PA USA

**Keywords:** Behavioural genetics, Population genetics, Drug discovery

## Abstract

Human genetic studies of smoking behavior have been thus far largely limited to common variants. Studying rare coding variants has the potential to identify drug targets. We performed an exome-wide association study of smoking phenotypes in up to 749,459 individuals and discovered a protective association in *CHRNB2*, encoding the β2 subunit of the α4β2 nicotine acetylcholine receptor. Rare predicted loss-of-function and likely deleterious missense variants in *CHRNB2* in aggregate were associated with a 35% decreased odds for smoking heavily (odds ratio (OR) = 0.65, confidence interval (CI) = 0.56–0.76, *P* = 1.9 × 10^−8^). An independent common variant association in the protective direction (rs2072659; OR = 0.96; CI = 0.94–0.98; *P* = 5.3 × 10^−6^) was also evident, suggesting an allelic series. Our findings in humans align with decades-old experimental observations in mice that β2 loss abolishes nicotine-mediated neuronal responses and attenuates nicotine self-administration. Our genetic discovery will inspire future drug designs targeting *CHRNB2* in the brain for the treatment of nicotine addiction.

## Abstract (web summary)

An exome-wide association study of six smoking phenotypes in up to 749,459 individuals identifies associations of rare coding variants in *CHRNB2* that may reduce the likelihood of smoking.

## Main

Tobacco smoking is one of the greatest hazards to human health, accounting for over 200 million disability-adjusted life years and 7 million deaths each year globally^1^. The currently available first-line smoking-cessation drugs (varenicline and bupropion) were introduced more than 2 decades ago, even before the Human Genome Project was completed and the genomic revolution started^2–4^. Despite their proven efficacy and wide usage^5^, smoking remains a global health hazard, warranting advancements in smoking-related drug-discovery efforts that make use of recent innovations in therapeutic design and delivery^6^.

Large-scale rare variant association studies have the potential to advance drug discovery^7–10^. Drug designs inspired by naturally occurring genetic variants that protect humans against diseases have been successful in the past, for example, inhibitors of the enzyme PCSK9 for the treatment of hypercholesterolemia^11–13^. Smoking behavior is strongly influenced by genetics, with twin-based heritability estimates ranging between 45% (for smoking initiation) and 75% (for nicotine dependence)^14^. Genetic variants across the entire minor allele-frequency (MAF) spectrum (common (MAF > 1%), low-frequency (MAF, 0.1–1%) and rare (MAF < 0.1%) variants) contribute to this high heritability^15^. However, human genetic studies of smoking behavior have thus far focused mainly on common and low-frequency variants (that can be imputed with at least moderate accuracy)^16–19^. Such genome-wide association studies (GWASs) were successful in identifying genomic regions associated with smoking. In contrast to GWASs, only a very few rare variant studies of smoking exist to date^15,20^. Although such studies have demonstrated that rare variants contribute substantially to smoking heritability, very few genes have been confidently linked to smoking based on rare variant associations^15,20^.

Unlike common variant associations, rare coding variant associations often pinpoint causal genes^21^, inform effect direction^21,22^, guide follow-up experiments^23^ and provide an estimate of the therapeutic efficacy^11,24^ and safety^25^ of targeting a gene or its product. Even for known drug targets, discovering human genetic evidence is valuable, as it can improve our understanding of the drug mechanisms and help develop new therapeutic modalities to treat diseases^26^. Hence, with the goal of discovering drug targets for smoking, we undertook a large-scale exome-wide association study (ExWAS) of smoking behavior involving up to 749,459 individuals. We studied the associations of rare coding variants in the human genome, captured via exome sequencing, with six major smoking phenotypes and a range of secondary phenotypes including smoking-related diseases. We also selectively explored the rare variant associations at the known GWAS loci and conducted ancestry-specific and cross-ancestry GWAS meta-analyses for the six smoking phenotypes to validate known loci and identify new loci. Finally, we studied the combined influences of both common and rare variants on smoking behavior.

## Results

### Exome-wide significant associations

**Fig. 1:**
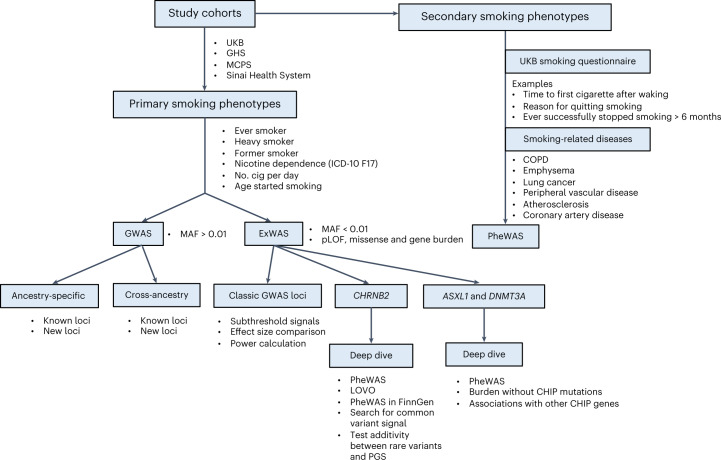
Study design. The flow chart summarizes the overall study design in terms of cohorts, phenotypes and types of genetic analyses performed. ICD, International Classification of Diseases.

**Fig. 2:**
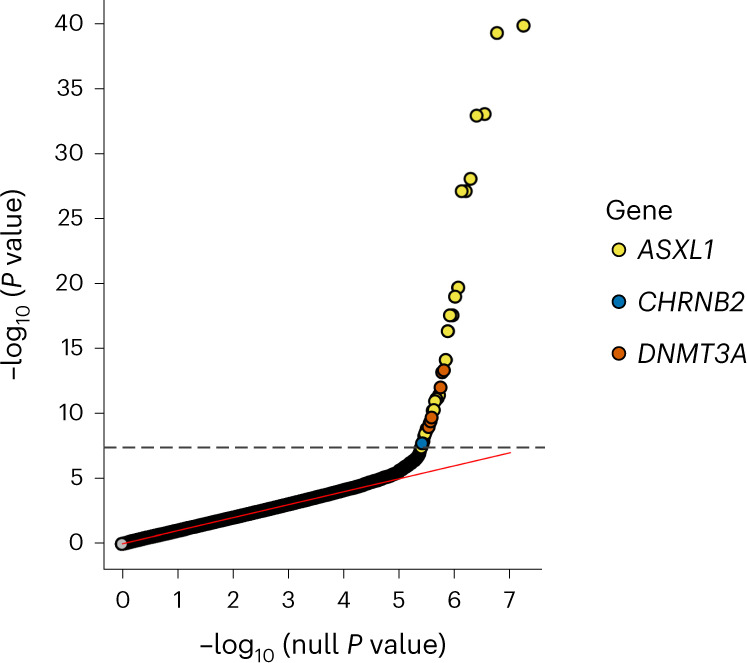
Discovery of rare variants associated with smoking phenotypes. Quantile–quantile (QQ) plot of the rare variant associations (both variant and burden associations) with six smoking phenotypes (ever smoker, heavy smoker, former smoker, nicotine dependence, cig per day and age started smoking). The dashed line corresponds to the exome-wide significant threshold, 4.5 × 10^−8^, determined based on a 1% FDR correction applied across all the associations (*n* tests = 8,417,987).

The overall study design is shown in Fig. 1. We performed ExWAS meta-analyses for six primary phenotypes (ever smoker, heavy smoker, former smoker, nicotine dependence, cigarettes smoked per day (cig per day) and age started smoking) in sample sizes ranging from 112,670 (cig per day) to 749,459 (ever smoker). The study cohorts and phenotype definitions are described in the Methods, and the cohort-specific sample sizes and participant demographics are summarized in Supplementary Tables 1 and 2, respectively. We focused on coding variants of two functional categories: missense variants and predicted loss-of-function (pLOF) variants (frameshift, splice donor, splice acceptor, stop lost, stop gain and start lost) with MAF < 0.01. In addition to variant-level associations, we also studied gene-level associations, using burden tests in which either pLOF variants only or pLOF and likely deleterious missense variants (that is, predicted to be deleterious by five different algorithms) in a gene are aggregated to create burden masks (or variant sets), which are then tested for association with the phenotypes (Methods)^21^. The burden masks were created using variants at five MAF thresholds (<0.01, <0.001, <0.0001, <0.00001 and singletons) (Supplementary Table 3). Altogether, we performed 8,417,987 association tests across six smoking phenotypes. Applying a false detection rate (FDR) of 1% (corresponding *P* value = 4.5 × 10^−8^), we identified 35 significant associations implicating three genes: *ASXL1*, *DNMT3A* and *CHRNB2* (Fig. 2, Supplementary Fig. 1 and Supplementary Table 4). Although these results were based on analyses in which individuals of all ancestries were pooled together, we found that the results were highly similar to those from a cross-ancestry meta-analysis or a meta-analysis involving only individuals of European ancestry, suggesting that the results were not influenced by population stratification (Supplementary Fig. 2).

### Associations of rare variants in *CHRNB2*

**Fig. 3:**
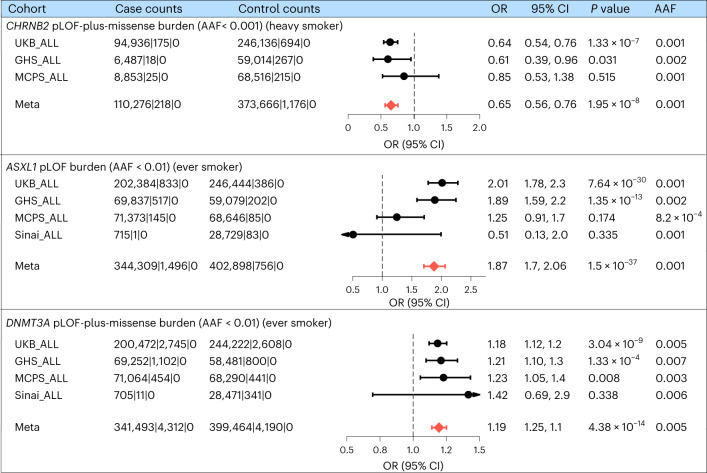
Forest plots of the top burden–trait associations of the significant genes. Cohort-level and meta-analysis summary statistics of the most significant burden–trait associations for each of the three exome-wide significant genes are summarized using forest plots. The ORs and 95% CIs are plotted. The columns ‘case counts’ and ‘control counts’ show the case and control sample sizes, respectively, broken down to the number of carriers of the homozygous reference, heterozygous and homozygous alternative genotypes. For burden definitions, refer to Supplementary Table 2. ALL, all ancestries; AAF, alternative allele frequency (combined frequency of all the variants aggregated in the burden mask).

**Extended Data Fig. 1:**
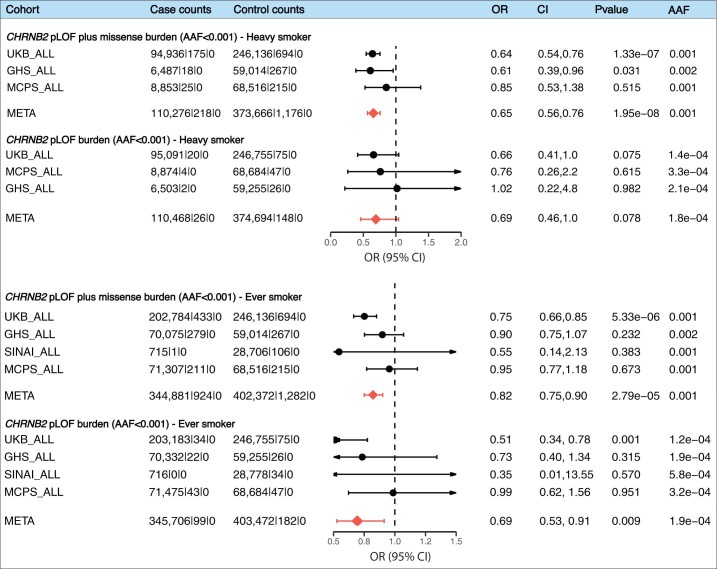
Forest plots of *CHRNB2* burden associations with *heavy-smoker* and *ever-smoker*. The forest plot displays the cohort-level and meta-analysis associations of the *CHRNB2* pLOF-only (AAF<0.001) and pLOF plus missense (AAF<0.001) burden masks with *heavy-smoker* and *ever-smoker* tested using REGENIE (Methods). The odds ratios and 95% confidence intervals are plotted. The columns ‘case counts’ and ‘control counts’ show the case and control sample sizes, respectively, broken down to the number of carriers of the homozygous reference, heterozygous and homozygous alternative genotypes.

The primary phenotype that discovered the *CHRNB2* association was heavy smoker, where cases were individuals who smoked at least ten cigarettes per day either currently or formerly (*n* = 110,494), and controls were individuals who have never smoked in their lifetime (*n* = 374,842). The strongest association was observed for pLOF-plus-missense burden (an aggregate of pLOF and likely deleterious missense variants in *CHRNB2* with MAF < 0.001), for which the odds of being a heavy smoker were significantly lower in carriers than in non-carriers (OR = 0.65; CI = 0.56–0.76; *P* = 1.9 × 10^−8^). The rare variant burden association was independent of any nearby common variant associations with *P* < 0.01 (Supplementary Fig. 3 and Methods), and the effect estimates were consistently in the protective direction across the three cohorts that contributed to the meta-analysis (Fig. 3). The protective association of *CHRNB2* pLOF-plus-missense burden with heavy smoking was observed irrespective of how we defined heavy smoking (Supplementary Fig. 4). Furthermore, the protective association was also seen for the ever smoker phenotype (where individuals who ever smoked regularly in their lifetime were defined as cases, *n* = 345,805) but was less significant than for the heavy smoker phenotype, despite a relatively larger sample size, highlighting the importance of phenotype specificity in gene discovery (Extended Data Fig. 1). However, when considering pLOF-only burden (an aggregate of pLOF variants in *CHRNB2* with MAF < 0.001), which provides the strongest evidence on the direction of the association, the association reached at least a nominal level of significance (*P* < 0.05) only for the ever smoker phenotype but not for the heavy smoker phenotype, likely because the ever smoker phenotype captured more pLOF carriers (281 carriers) than the heavy smoker phenotype (174 carriers), suggesting that a larger sample size at the expense of phenotype specificity is also valuable, particularly at the rarer end of the allele-frequency spectrum.

**Extended Data Fig. 2:**
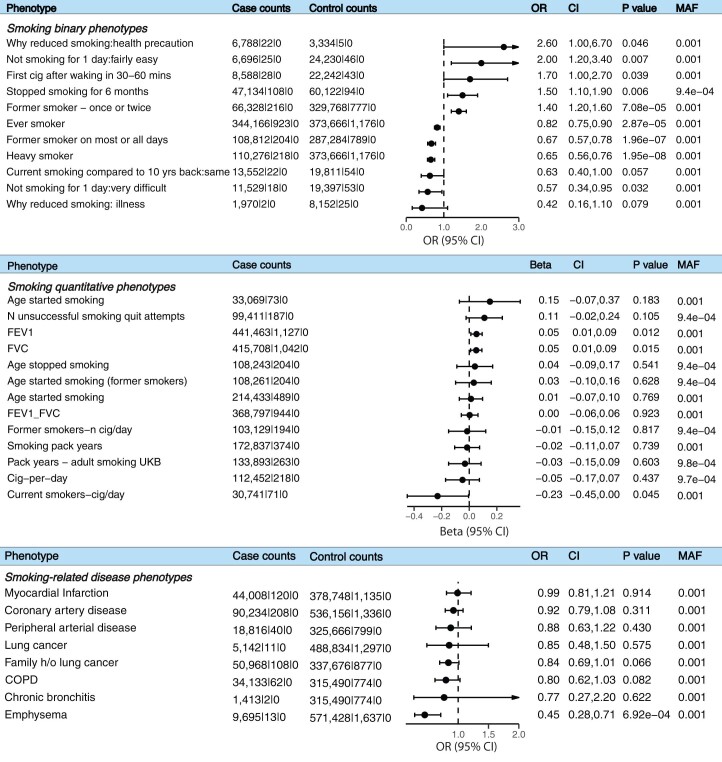
Forest plots of *CHRNB2* burden associations with secondary smoking phenotypes. The forest plots display the cohort-level or meta-analysis associations of *CHRNB2* pLOF plus missense (AAF<0.001) burden mask with binary (P<0.1) and quantitative smoking phenotypes (major smoking phenotypes and phenotypes derived based on UKB lifestyle questionnaire) and smoking-related diseases tested using REGENIE (Methods). The odds ratios (or Beta estimates) and 95% confidence intervals are plotted. The columns ‘case counts’ and ‘control counts’ show the case and control sample sizes, respectively, broken down to the number of carriers of the homozygous reference, heterozygous and homozygous alternative genotypes. FEV1 – Forced expiratory volume in 1 sec; FVC – Forced vital capacity; FEV1_FVC – FEV1:FVC ratio; COPD – Chronic obstructive pulmonary disease.

We next studied the association of *CHRNB2* pLOF-plus-missense burden with a range of secondary smoking phenotypes, mainly derived from UK Biobank (UKB)^27^ participants’ responses to a lifestyle questionnaire related to smoking (Methods). The overall association pattern was in line with our main finding that rare pLOF and likely deleterious missense variants in *CHRNB2* in aggregate confer protection against smoking addiction (Extended Data Fig. 2 and Supplementary Table 5). We also studied the burden associations with a curated list of binary and quantitative health phenotypes related to smoking and observed suggestive associations, all in the protective direction, for example, emphysema (OR = 0.45; CI = 0.28–0.71; *P* = 6.9 × 10^−4^), chronic obstructive pulmonary disease (COPD; OR = 0.80; CI = 0.62–1.03; *P* = 0.08) and family history of lung cancer (OR = 0.84; CI = 0.69–1.01; *P* = 0.06) (Extended Data Fig. 2).

**Fig. 4:**
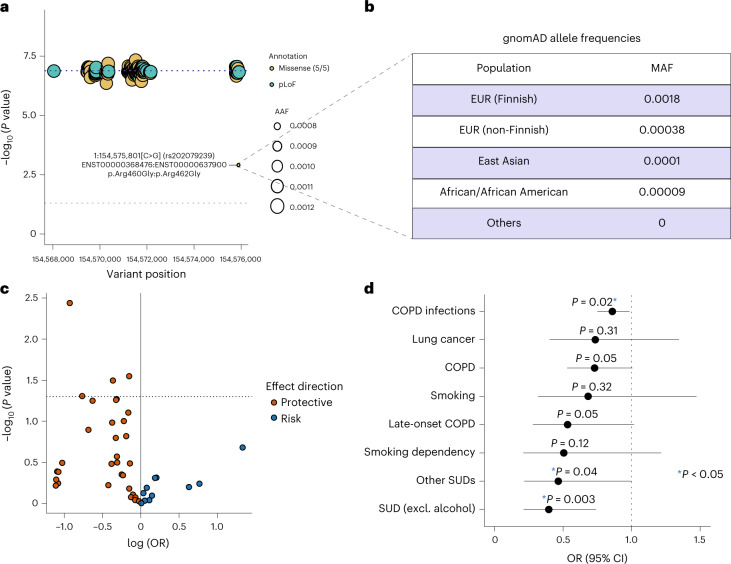
A Finnish-enriched missense variant contributes most to the *CHRNB2* burden association. **a**, Results from LOVO analysis (Methods) of the *CHRNB2* pLOF-plus-missense burden (AAF < 0.001) in the UKB. The LOVO *P* values are plotted against the variant positions. The dashed blue line corresponds to the *P* value of the full burden association. The dashed gray line corresponds to *P* = 0.05. **b**, MAFs of Arg460Gly (rs202079239) in different populations in the gnomAD database. EUR, European ancestry. **c**, Volcano plot showing the PheWAS associations of Arg460Gly with smoking-related phenotypes in the FinnGen database. The dashed line corresponds to *P* = 0.05**. d**, ORs and 95% CIs of selected phenotype associations of Arg460Gly in the FinnGen database are displayed. Excl., excluding, SUD, substance use disorder.

No individual pLOF or missense variants in *CHRNB2* surpassed the study-wide significance threshold, suggesting that our sample sizes were still underpowered to capture single-variant associations. Using a leave-one-variant-out (LOVO) burden analysis^28^ (Methods), we identified a missense variant (rs202079239, Arg460Gly) that contributed the most to the pLOF-plus-missense burden association in the UKB (Fig. 4a and Supplementary Table 6). Importantly, even after excluding Arg460Gly, the burden association was still nominally significant with a protective OR (OR = 0.71; CI = 0.57–0.88; *P* = 0.001), suggesting that other variants in the burden mask also contributed to the association (Supplementary Table 6). Additionally, the Arg460Gly variant independently showed a moderately significant protective association with the heavy smoker phenotype (OR = 0.56; CI = 0.43–0.72; *P* = 1.1 × 10^−5^). We found that this variant has drifted to a higher frequency in Finns (gnomAD^29^ MAF = 0.0018) compared to non-Finnish Europeans (gnomAD MAF = 0.00038; Fig. 4b). Statistical power increases with MAF; hence we expected that the protective association of Arg460Gly with smoking or related phenotypes might be detectable in FinnGen^30^, a population-based cohort in Finland, despite its sample size being smaller than that of the UKB. A selective exploration of Arg460Gly with smoking, substance use and smoking-related lung disease phenotypes in the publicly available data from the FinnGen research project (freeze version 7) revealed significant enrichment for protective associations (hypergeometric test for enrichment, *P* = 0.03; Fig. 4c,d and Supplementary Table 7). At least two phenotypes showed nominally significant (*P* < 0.05) protective associations: substance-use disorder (excluding alcohol) (OR = 0.39; CI = 0.21–0.73; *P* = 0.003) and COPD (OR = 0.69; CI = 0.49–0.96; *P* = 0.03). Therefore, by exploiting the natural phenomenon of genetic drift in an isolated population^30^, we were able to validate the protective association of *CHRNB2* with smoking-related phenotypes in an independent cohort.

### Associations of common variants near *CHRNB2*

**Fig. 5:**
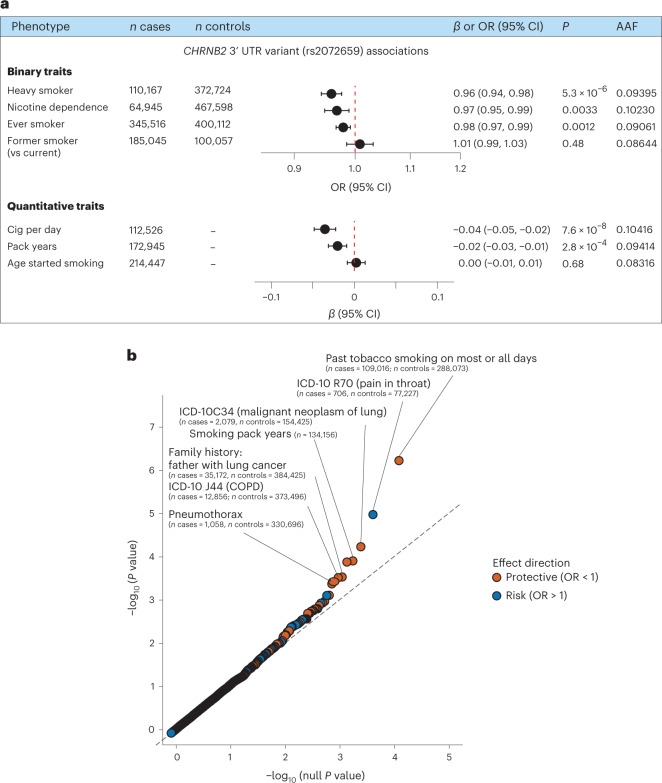
Association of a common 3′ UTR variant with smoking. **a**, Forest plots of associations of the *CHRNB2* 3′ UTR variant (rs2072659) with the major smoking phenotypes based on cross-ancestry meta-analyses (Methods); either ORs (if binary traits) or *β* estimates (in s.d. units) and their 95% CIs are plotted. **b**, QQ plot of the PheWAS associations of rs2072659 in the UKB and the GHS cohorts.

Common variant associations by themselves often do not pinpoint the causal gene(s); when they do, they mostly bring limited insights into the druggability of the gene. However, when interpreted along with rare coding variant associations, they can offer valuable insights. To this end, we searched for any known common variant GWAS signals near *CHRNB2* that were reported previously for smoking-related traits. Liu et al.^16^ have reported a GWAS association with cig per day near *CHRNB2* where the fine-mapped 95% credible set contained a single variant, rs2072659, located within the 3′ untranslated region (UTR) of *CHRNB2*. This variant showed significant (*P* < 0.05) associations in our dataset with multiple smoking phenotypes including heavy smoker (OR = 0.96; CI = 0.94–0.98; *P* = 5.3 × 10^−6^), all in the protective direction (Fig. 5a). In a phenome-wide association study (PheWAS) of this variant across 7,469 phenotypes in two of the large cohorts (UKB and Geisinger Health System (GHS)), the strongest association was with smoking (Fig. 5b). In addition, seven of the top ten associations were with smoking-related phenotypes, all in the protective direction.

### Associations of clonal hematopoiesis of indeterminate potential mutations in *ASXL1* and *DNMT3A*

**Fig. 6:**
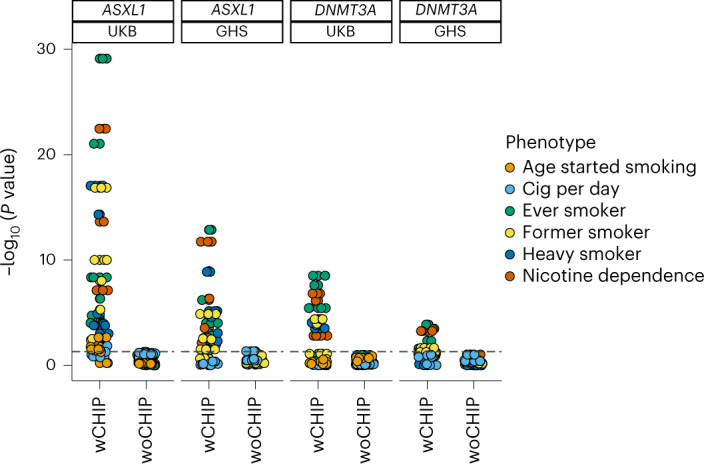
Association of *ASXL1* and *DNMT3A* CHIP mutations with smoking. We constructed pLOF-only and pLOF-plus-missense burden masks at five allele frequency thresholds using all variants (wCHIP) and excluding CHIP variants (woCHIP) in the UKB and the GHS cohorts and tested their associations with the six major smoking phenotypes using REGENIE (Methods). The burden association *P* values are plotted, and the summary statistics including sample sizes are provided in Supplementary Table 8. The dashed line corresponds to the significance threshold after adjusting for multiple testing (1% FDR correction).

**Extended Data Fig. 3:**
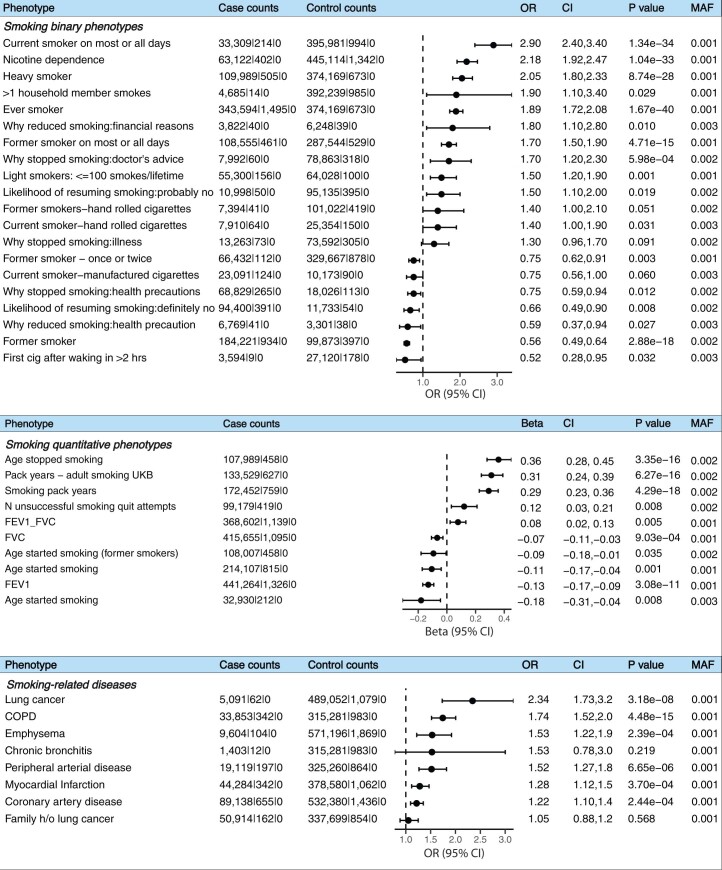
Forest plots of *ASXL1* burden associations with secondary smoking phenotypes. The forest plots display the cohort-level or meta-analysis associations of *ASXL1* pLOF only burden mask (AAF<0.01) with binary and quantitative smoking phenotypes (major smoking phenotypes and phenotypes derived based on UKB lifestyle questionnaire with P<0.1) and smoking-related diseases tested using REGENIE (Methods). The odds ratios (or beta estimates) and 95% confidence intervals are plotted. The columns ‘case counts’ and ‘control counts’ show the case and control sample sizes, respectively, broken down to the number of carriers of the homozygous reference, heterozygous and homozygous alternative genotypes.

**Extended Data Fig. 4:**
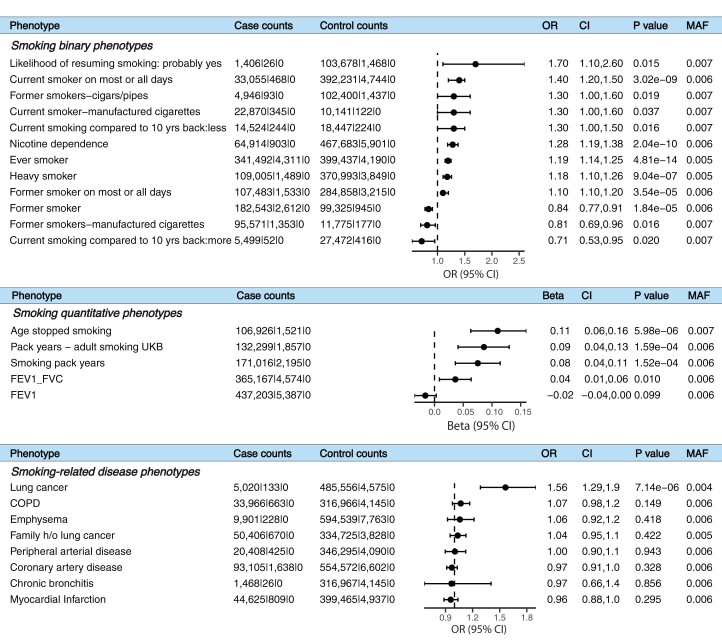
Forest plots of *DNMT3A* burden associations with secondary smoking phenotypes. The forest plots display the cohort-level or meta-analysis associations of *DNMT3A* pLOF plus missense burden mask (AAF<0.01) with binary and quantitative smoking phenotypes (major smoking phenotypes and phenotypes derived based on the UKB lifestyle questionnaire) and smoking-related diseases tested using REGENIE (Methods). The odds ratios (or beta estimates) and 95% confidence intervals are plotted. The columns ‘case counts’ and ‘control counts’ show the case and control sample sizes, respectively, broken down to the number of carriers of the homozygous reference, heterozygous and homozygous alternative genotypes.

**Extended Data Fig. 5:**
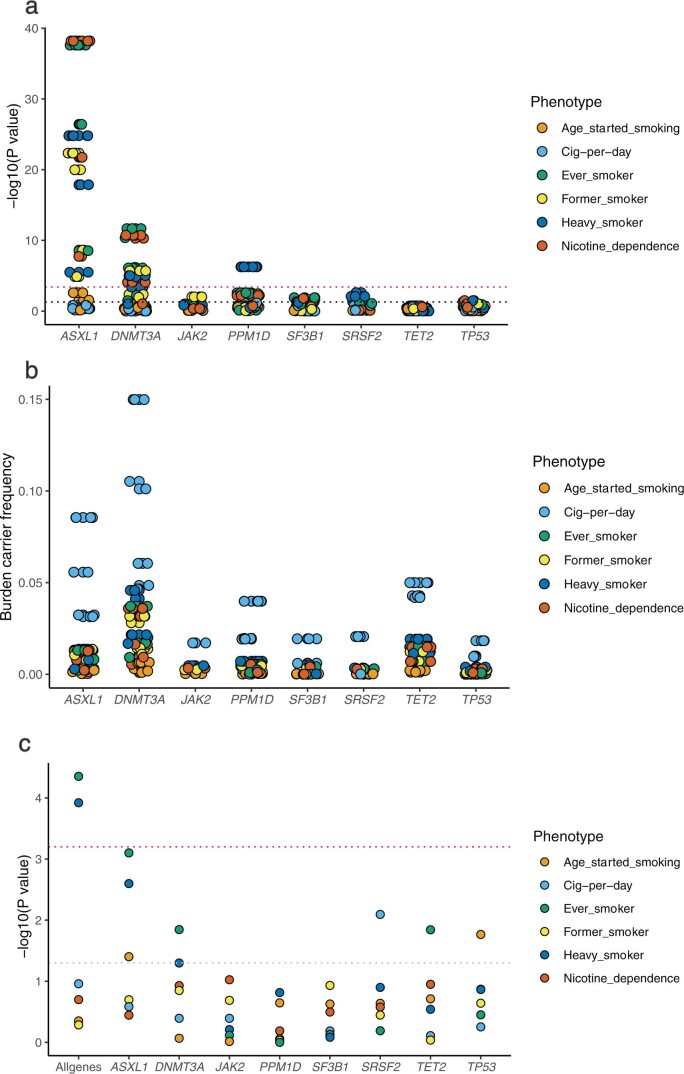
Associations of CHIP mutations with smoking. **a**. pLOF only and pLOF plus missense burden masks for eight recurrent CHIP genes were created in the UKB and GHS cohorts by aggregating only high-confident CHIP mutations (Methods) and tested for their associations with the six smoking phenotypes. The results were meta-analyzed between the GHS and UKB cohorts and the resulting P values are plotted. The dotted red line corresponds to FDR 1% P value threshold and the black dotted line corresponds to P = 0.05. **b**. The alternative allele frequencies (AAF) of the burden masks (combined AAF of all the variants aggregated in a mask) are plotted. **c**. Variant allele fractions (VAF) of CHIP mutations in the eight most recurrent CHIP genes were aggregated gene-wise and all together in the CHIP carriers in the UKB and GHS cohorts (when the same individual carried more than one CHIP mutation, we took the average of the VAF) and tested for associations with the six smoking phenotypes. The UKB and GHS combined association P values are plotted. The red dotted line corresponds to FDR 1% P value and the black dotted line corresponds to P = 0.05.

Among the three exome-wide significant genes, *ASXL1* and *DNMT3A* showed the strongest associations with most of the smoking phenotypes (Figs. 2 and 3, Extended Data Figs. 3 and 4 and Supplementary Tables 4 and 5). However, both *ASXL1* and *DNMT3A* are known to accumulate somatic mutations in circulating blood cells with increasing age in the general population, a phenomenon described as clonal hematopoiesis of indeterminate potential (CHIP)^31^. When the DNA source for exome sequencing is peripheral blood, standard exome variant-calling workflows capture CHIP mutations along with germline variants^32,33^. We have previously reported a comprehensive genetic analysis of CHIP, in which we systematically called somatic variants in participants of the UKB and the GHS cohorts and studied their germline associations^33^. It is well known that smoking is strongly associated with CHIP^34,35^, and the association of *ASXL1* CHIP mutations with smoking in the UKB has been previously reported^35^. Hence, we were not surprised to learn that the *ASXL1* and *DNMT3A* associations were driven by CHIP mutations, which we confirmed through burden analyses based on burden masks with and without CHIP mutations and association analyses of the variant allele fraction (VAF) of CHIP mutations with smoking phenotypes (Fig. 6, Extended Data Fig. 5 and Supplementary Note). As was previously proposed, the association of CHIP mutations with smoking phenotypes suggests that smoking offers a clonal advantage to certain CHIP mutations, although the underlying mechanisms have yet to be understood. Also, our findings echo the caution previously raised by many in relation to using exome-sequencing data based on blood samples to establish genetic diagnoses for Mendelian diseases in adults^36,37^ (Supplementary Note).

### Association of rare variants at known GWAS loci

**Extended Data Fig. 6:**
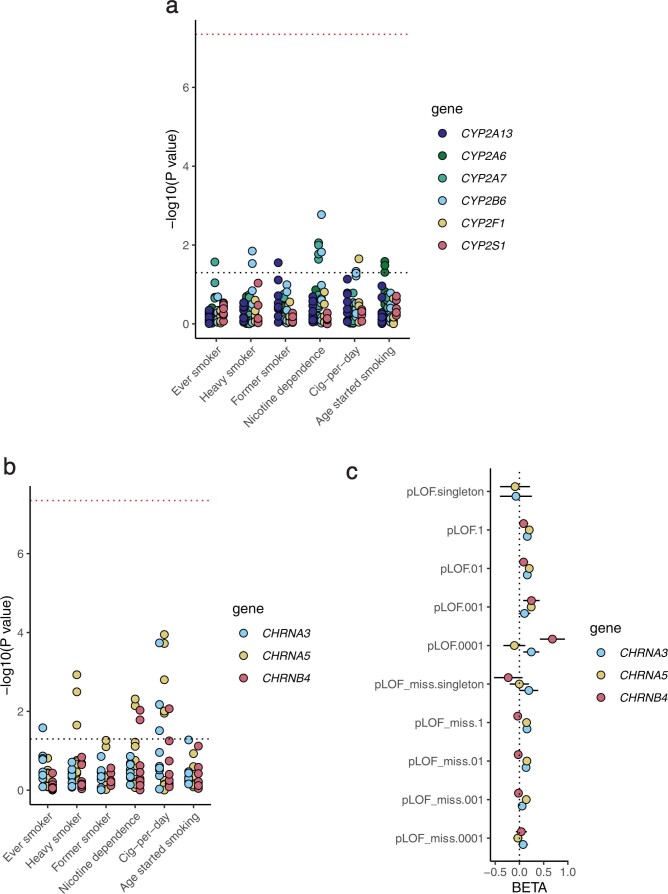
Rare variant associations at the classic *CYP2A6* and *CHRNA5* GWAS loci. **a**. P values of the pLOF only and pLOF plus missense burden associations of cytochrome gene cluster at the *CYP2A6* GWAS locus with the six smoking phenotypes are plotted. The red dotted line corresponds to FDR 1% P value and the black dotted line corresponds to P = 0.05. **b**. P values of the pLOF only and pLOF plus missense burden associations of nicotine acetylcholine receptor (nAChR) genes at the *CHRNA5* GWAS locus with the six smoking phenotypes are plotted. The red dotted line corresponds to FDR 1% P value and the black dotted line corresponds to P = 0.05. **c**. The beta estimates (in SD units) and 95% confidence intervals of the nAChR burden associations with cig-per-day (N = 112,670) are plotted. The sample sizes of the associations shown in panels a, b, and c are provided in Supplementary Table 11.

**Extended Data Fig. 7:**
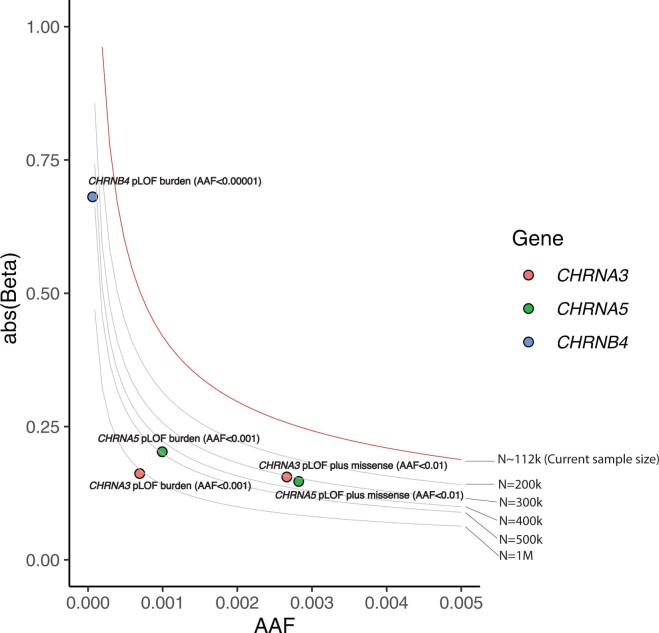
Power calculations for rare variant discovery at the *CHRNA5* GWAS locus. Assuming an 80% power and P value of 5e-8, detectable effect sizes at various minor allele frequency values were calculated for the current sample size of cig-per-day (the smoking trait most associated with *CHRNA5* locus) as well for a series of sample sizes up to 1 million. The observed effect sizes for pLOF only burden and pLOF and missense burden associations of *CHRNA5*, *CHRNA3* and *CHRNB4* are plotted; all the points lay below the red line, which marks the detection limit of our current sample size, suggesting that we are underpowered. Based on the intersections of the grey lines with the points marking the observed effect sizes, we can approximately guess what sample size will be required to detect these burden signals at P value 5e-8.

Two of the strongest genetic risk loci for smoking that were identified early in the GWAS timeline were locus 15q25.1, containing three nicotine acetylcholine receptor (nAChR) genes (*CHRNA5*, *CHRNA3* and *CHRNB4*)^38,39^, and locus 19q13.2, containing a cluster of cytochrome P450 enzyme-coding genes (CYP2A, CYP2B and CYP2F subfamilies); both strongly influence the number of cigarettes smoked per day^40,41^. Although none of the genes were significant at the exome-wide level in our analysis, given their strong biological links to smoking, we explored these loci for evidence of any subthreshold rare variant associations. At the cytochrome P450 locus, we found little evidence for rare variant associations beyond the known common variant signals (Extended Data Fig. 6a and Supplementary Table 11). However, we observed nominal rare variant gene burden associations with cig per day at locus 15q25.1, implicating all three nAChRs (*CHRNA5*, *CHRNA3* and *CHRNB4*) with effect sizes larger than those observed for common variants (Extended Data Fig. 6b). Notably, the largest effect size was observed for the *CHRNB4* pLOF-only rare variant burden, where the 13 pLOF carriers smoked on average ~6.8 cigarettes per day more than non-carriers (*β* = 0.68 s.d.; CI = 0.17–1.18; *P* = 0.008; Extended Data Fig. 6c). This effect size is approximately three to four times larger than the largest effect sizes observed for *CHRNA5* (*β* = 0.23; CI = 0.05–0.40; *P* = 0.01) and *CHRNA3* (*β* = 0.16; CI = 0.02–0.31; *P* = 0.03) pLOF-only rare variant burden and ~7.5 times larger than that for rs16969968 (approximately one cigarette more; *β* = 0.09; CI = 0.09–0.10; *P* = 3.8 × 10^−125^), a well-characterized common risk variant at this locus (Supplementary Table 11). Power calculations based on observed effect sizes suggest that these associations will likely emerge as significant at the genome-wide level when the sample size for ExWAS of the cig per day phenotype reaches between 300,000 and 500,000 (Extended Data Fig. 7).

**Extended Data Fig. 8:**
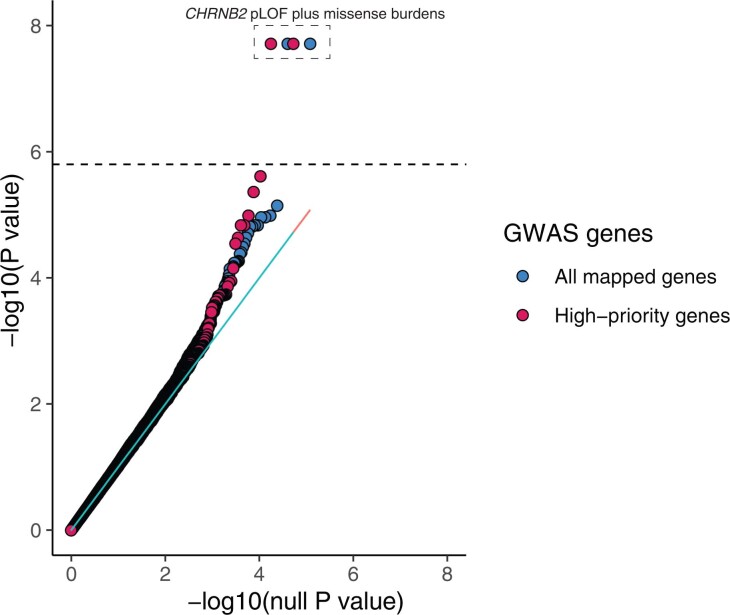
Association of rare variant burden in genes at the GWAS loci associated with smoking behavior. Rare pLOF only and pLOF and missense burden associations were tested focusing only on the genes located at the known GWAS loci identified by the recent largest GWAS of smoking to date. We studied two gene lists prioritized by Saunders et al.^19^: a list of genes mapped to all the identified GWAS loci and a list of ‘high-priority genes’ mapped to GWAS loci with less than five fine-mapped variants. QQ plots of the meta-analysis P values of burden associations are shown. The dashed line corresponds to FDR 1% P value threshold.

Previous exome studies have shown that rare variant associations are enriched near GWAS loci for many human diseases and traits^21,42^. Hence, we analyzed the burden associations, focusing only on genes mapped to GWAS loci^19^ (Methods). We observed no significant rare variant burden associations other than the association of *CHRNB2* pLOF-plus-missense burden with the heavy smoker phenotype (Extended Data Fig. 8). The results suggested that our current sample sizes are underpowered to capture the convergence between common and rare variant associations at the known smoking GWAS loci.

### Cross-ancestry and ancestry-specific GWAS

We first performed GWAS for the six primary smoking phenotypes in individuals of European ancestries and used these results to analyze SNP-based heritability (SNP-*h*^2^) and genetic correlations using a European ancestry-based linkage disequilibrium (LD) reference panel^43^. Our SNP-*h*^2^ estimates were comparable to previously reported estimates^16^ (Supplementary Fig. 5a and Supplementary Table 12). Also, our GWAS results showed strong genetic correlations with the previous GWAS results^16^ (Supplementary Fig. 5b and Supplementary Table 13), which suggests high reproducibility of the polygenic signals of the studied smoking phenotypes. Also, we observed moderate-to-large genetic correlations across our six phenotypes, suggesting shared genetic architecture across the phenotypes (Supplementary Fig. 5c and Supplementary Table 14).

Next, we performed cross-ancestry GWAS meta-analyses for the six primary smoking phenotypes. Across all the phenotypes, in total, we identified 328 LD-independent loci, of which a majority (94%) are known. This was expected, given that a GWAS with a much larger sample size has been published before^16^ (Supplementary Fig. 6a–f and Supplementary Table 16). Among the new loci, an X chromosome locus that we identified for nicotine dependence deserves special mention, as it implicates a nicotinic receptor-related gene. This locus, Xq22.1, harbors *TMEM35A* (the closest gene to the index variant), also referred to as *NACHO* (new acetylcholine receptor chaperone); this gene encodes a molecular chaperone protein that is involved in the assembly of α7, α6β2 and α6β2β3 nAChRs^44^. Mice lacking *Tmem35a* develop hyperalgesia^44^, and we observed that the index variant at this locus is also associated with increased intake of oxycodone, an analgesic medication, in the UKB (OR = 1.58; *P* = 0.0001; data from https://www.opentargets.org)^45^, suggesting that this locus might influence both smoking and pain phenotypes in humans.

After European ancestries, the second largest proportion (19%) of our study participants were of admixed American ancestries (AMR), mostly from the Mexico City Prospective Study (MCPS) cohort^46^. Published GWASs of smoking behavior in AMR ancestries are sparse^47^. In the AMR-specific GWAS, we identified 25 independent loci across the six phenotypes, of which 15 are known and 10 are new (Supplementary Table 16). The known loci include some of the strongest GWAS loci identified in European-specific GWAS: *CHRNA5* (ref. ^39^), *CHRNA4* (ref. ^48^), *DBH*^41^, *CYP2A6* (refs. ^40,41^) and *NCAM1* (ref. ^49^) (Supplementary Table 16). In AMR ancestries, we also identified an X chromosome locus that has been previously linked to smoking in those of European ancestries^18^. Notably, at this locus (with *GPR101* in the vicinity), we identified a genome-wide significant association with the heavy smoker phenotype in the AMR-specific GWAS (rs1190734; OR_AMR_ = 0.83 (0.79–0.88); *P*_AMR_ = 1.2 × 10^−11^) but only a nominal association with the heavy smoker phenotype in the European-specific GWAS (OR_EUR_ = 0.98 (0.97–0.99); *P*_EUR_ = 0.001). However, the same variant showed genome-wide significant association with the cig per day phenotype in European-specific GWAS (*β*_EUR_ = −0.02; *P*_EUR_ = 7.6 × 10^−16^), corroborating the GWAS signal at this locus reported previously for the cig per day phenotype^18^. Whether this locus is associated with the cig per day phenotype in AMR ancestry with a larger effect size than that in European ancestry is not clear, as we did not have this phenotype in the MCPS cohort at the time of this analysis. Nevertheless, the findings overall suggest that the *GPR101* locus influences smoking behavior in both European and AMR ancestries. Regarding the ten new loci identified in the AMR ancestries, as expected, many (seven loci) harbored variants that are relatively more common in AMR ancestries than in European ancestries, thereby offering higher statistical power for discovery; for example, at 10q21.1, an intergenic locus, we identified a genome-wide significant association with the heavy smoker phenotype where the index variant is observed in ~10% of admixed Americans but only in ~0.05% of Europeans; at 8p22 (closest gene, *C8orf48*), we identified a genome-wide significant association with the ever smoker phenotype, where the index variant is observed in ~30% of admixed Americans but only in ~7% of Europeans (Supplementary Table 16).

### Interplay between common and rare variants

**Fig. 7:**
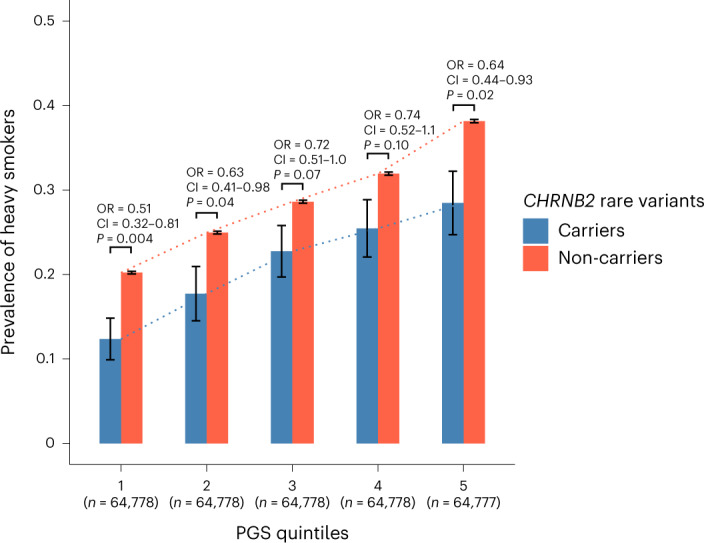
Additive effects between *CHRNB2* rare variants and smoking PGS. Prevalence estimates of heavy smokers among *CHRNB2* rare variant carriers and non-carriers within each of the five PGS quintiles in the UKB are plotted. Standard errors of the prevalence estimates, displayed as error bars, were calculated using the formula √(*pq*/*n*), where *n* is the number of individuals in each group, *p* is the prevalence of heavy smoker in the group and *q* is 1 − *p*. The PGS was based on a GWAS meta-analysis of the ever smoker phenotype (Methods). *CHRNB2* rare variants are those that were aggregated into the *CHRNB2* pLOF-plus-missense (AAF < 0.001) burden mask. Statistical differences in the prevalence between carriers and non-carriers were tested using a logistic regression analysis within each quintile; ORs, 95% CIs and *P* values are shown.

Large-scale sequencing projects provide increased power to detect additive effects between common and rare variants for many diseases and traits. For example, we have previously demonstrated an additive effect between *GPR75* obesity-protective rare variants and polygenic score (PGS) for obesity based on common variants^10^. We performed a similar analysis to test whether an additive effect is also evident for *CHRNB2* rare variants and smoking PGS. We calculated smoking PGS for UKB participants of European ancestries based on a GWAS of the ever smoker phenotype performed in an independent sample (a meta-analysis of GWAS and Sequencing Consortium of Alcohol and Nicotine use (GSCAN) GWAS^19^ results excluding 23andMe and the UKB with the GWAS results of the GHS^50^, one of our largest European cohorts). First, we studied the associations of *CHRNB2* pLOF-plus-missense burden and smoking PGS with heavy smoking within a single regression model that included an interaction term between the burden mask and the PGS (Methods). Both burden mask (OR = 0.66; 95% CI = 0.56–0.79; *P* = 3.4 × 10^−6^) and the PGS (*β* = 0.33; standard error (SE) = 0.004; *P* = 1 × 10^−300^) were associated with heavy smoking without a statistically significant interaction (*P* = 0.71). The results suggest that rare variants and the PGS influence the risk of heavy smoking independently. Second, to demonstrate the additive effect, we binned UKB individuals into quintiles based on their smoking PGS and quantified the prevalence of heavy smokers in *CHRNB2* pLOF-plus-missense burden mask carriers (the burden mask that showed the strongest association with the heavy smoker phenotype) and non-carriers. The prevalence of heavy smokers increased in both carriers and non-carriers from lower to higher PGS quintiles (Fig. 7 and Supplementary Table 17). Importantly, within each of the quintiles, the prevalence of heavy smokers was lower in *CHRNB2* rare variant carriers than in non-carriers, demonstrating an additive effect between PGS and rare variants. The additivity implies that the smoking PGS modifies the penetrance of *CHRNB2* rare variants and vice versa, that is, the protective effect of *CHRNB2* rare variant burden is attenuated in individuals with higher PGS compared to in individuals with lower PGS, and the risk effect of increased PGS is attenuated in rare variant carriers compared to in non-carriers.

## Discussion

**Extended Data Fig. 9:**
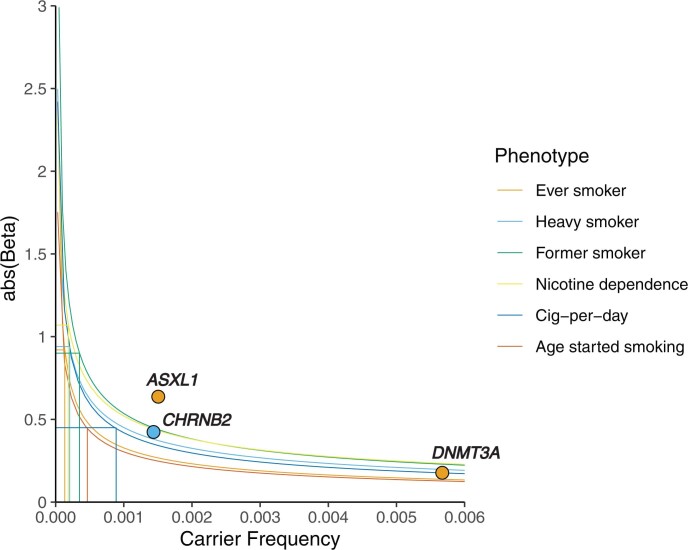
Power calculations for gene discovery using the current sample size. Assuming an 80% power, P value threshold of 4e-8 (exome-wide significant threshold of the current study based on FDR 1%), effect sizes (that is, beta values) were computed for a range of minor allele frequencies (combined allele frequency in case of burden masks) for a given sample size (varies across phenotypes). The computed effect sizes (absolute values of beta estimates) are plotted against minor allele frequencies (carrier frequency) for six smoking phenotypes. The carrier frequency corresponding to 100 carriers, calculated for each of the phenotype based on the corresponding sample size, in the X axis and the corresponding effect size in the Y axis are marked with straight lines. The top association of the three genes identified as exome-wide significant are plotted with the color corresponding to the associated phenotype. Based on these power curves, we had 80% power to detect any variant or burden associations with ever-smoker, heavy-smoker and /former-smoker/ with odds ratio ~2.5 or higher (0.4 or lower) when there are at least 100 carriers. And we had 80% power to detect any variant or burden associations with cig-per-day and age started smoking with beta 0.45 (equivalent to 4.7 extra cigarettes for cig-per-day and 1.9 yr earlier age for age started smoking) when there are at least 100 carriers. These calculations assume that there is no heterogeneity in the effect sizes across the cohorts, which is never the case for complex traits such as smoking. Hence, these estimates should be considered arbitrary. Importantly, the effect sizes for protective associations with binary phenotypes are likely overestimated due to imbalances in the case-control ratios.

GWASs of smoking behavior^16–19^ based on common variants have made tremendous progress in the field, with the recent GWAS involving more than 3 million individuals^19^. Such studies have substantially improved our understanding of the polygenic architecture of smoking phenotypes and have highlighted genes and pathways including nAChRs, genes involved in nicotine metabolism and dopaminergic and glutamatergic signaling^16^. However, to date, few studies based on whole-exome- or whole-genome-sequencing data have been reported^15,20^, and they involved sample sizes insufficient to capture associations at variant- and gene-level resolutions. Hence, our understanding of the contributions of rare variants to smoking behavior has been minimal thus far. In the present study, we performed a large-scale rare variant analysis in sample sizes that had enough power to identify associations of a rare variant or an aggregate of rare variants with an OR of 2.5 and above (or 0.4 and below) when there are at least 100 carriers (Extended Data Fig. 9). The fact that our analysis revealed only one germline association indicates that there are no ‘low-hanging fruits’ for smoking in the rare variant space other than *CHRNB2*. However, we acknowledge that this interpretation applies only to European populations, and we cannot exclude the possibility that rare variants exist that are more frequent in other ancestries and might be discovered in the future in similar or even smaller sample sizes than ours. Nevertheless, we note that 25% of our samples represent non-European ancestries, with the largest proportion (19%) representing admixed Americans^46^. However, the sample sizes, when broken down into individual ancestry groups, are still smaller than what would be necessary to make rare variant discoveries.

The major finding from our analysis is that individuals with rare pLOF and likely deleterious missense variants in *CHRNB2* are at decreased odds of smoking heavily. Although the top association was observed for the gene burden that combined both pLOF and missense variants, the concordant protective effect sizes observed for the pLOF-only burden strengthened our interpretation that what we observe is a loss-of-function association. This knowledge is crucial as it informs therapeutic hypotheses for drug design. Moreover, we identified a single deleterious missense variant that drifted to a higher frequency in the Finnish population, which gave us an opportunity to validate the protective associations in the FinnGen study^30^. The finding highlights the value of isolated populations to inform drug target discovery^51^.

Another important finding is the convergence of rare and common variant findings of *CHRNB2*. We highlight a common 3′ UTR variant, reported in previous GWASs^16,19^, that shows protective associations with multiple smoking phenotypes, suggesting that this variant likely decreases *CHRNB2* expression. Importantly, the OR of the common variant association with the heavy smoker phenotype was 0.96 as opposed to 0.65 for the pLOF-plus-missense rare variant burden. The pattern suggests a dose–response relationship between the gene and the phenotype in which varying levels of gene perturbations result in proportional effects on the phenotype. We particularly highlight the fact that this variant, although discovered in the earlier GWAS^16^, did not receive attention, as it was buried underneath the hundreds of GWAS associations, reflecting an important limitation of interpreting common variant findings. However, when interpreted in the light of rare variant findings, the common variant association stood out as highly valuable, exemplifying the combined value of GWAS and ExWAS in drug target discovery. Such observations will become frequent in the future with the rapidly growing population-scale ExWAS of human diseases and traits^52^.

*CHRNB2* codes for the β2 subunit of the α4β2 nAChR, which is the predominant nicotinic receptor expressed in the human brain^53^. The role of α4β2 nAChR in mediating nicotine effects has been well characterized by decades of animal studies^54,55^, thanks to the pioneering work of Picciotto and colleagues who demonstrated in 1995 that deletion of the gene encoding β2 in mice abolished nicotine-mediated effects on avoidance learning and reinforcement behavior^56,57^. However, we describe human genetic evidence supporting the hypothesis that loss of *CHRNB2* protects against nicotine addiction. Importantly, the protein encoded by *CHRNB2* can be viewed as a known drug target as it is a component of the α4β2 nAChR, which, being the major nicotine receptor in the brain, has been the target of most nAChR partial agonists and antagonists developed thus far, including cytisine (an α4β2 partial agonist^58^) and varenicline (an α4β2 partial agonist and antagonist^3^). Varenicline is the current drug of choice to aid smoking cessation and was developed in 1997 by Pfizer based on the molecular structure of cytisine^2,3^. In addition to α4β2, varenicline binds to various other nAChRs in the brain including α7, α3β4 and α6β2 (ref. ^59^). Given the established role of α4β2 in mediating rewarding and reinforcement actions of nicotine, it is believed that the α4β2-antagonistic action of varenicline helps with smoking cessation^3^. Our finding aligns with this hypothesis, emphasizing that human genetics is useful not only to discover new drugs but also to better understand the mechanism of action of old drugs that have been in use for decades, and such knowledge can pave the way for better drug designs with greater efficacy and limited adverse effects.

Limitations of our study include small sample sizes for finer quantitative phenotypes such as cig per day, which have limited our power to capture rare variant associations of genes mediating aversive effects of nicotine (for example, *CHRNA5*) and those related to nicotine metabolism (for example, *CYP2A6*)^39,40^. As is often the case, individuals of non-European ancestries were under-represented in our study cohorts, which has limited the generalizability of the findings to all ancestries^60,61^. However, we involved a substantial number of individuals of AMR ancestries, who belong to one of the most under-represented populations in human genetic studies, a step in the right direction^46^. With growing awareness of the importance of diversity in human genetic studies, the representation of non-European ancestries is expected to improve in future studies^60,61^. Finally, we have focused only on the coding regions of the genome captured via whole-exome sequencing, and therefore we may have missed rare variants with large effects on smoking behavior residing in noncoding regulatory regions. With the recent increase in large-scale whole-genome-sequencing efforts, rare large-effect regulatory variants influencing human diseases and traits are being discovered, and such discoveries may have the potential to lead to drug targets^62^. However, the question of whether whole-genome sequencing is a more cost-effective investment than whole-exome sequencing for drug target discovery has yet to be answered.

To conclude, we have performed a large-scale ExWAS of smoking behavior and identified a protective association between rare coding variants in *CHRNB2* and smoking. The results align with the findings from published knockout animal models and the mechanism of action of varenicline that is currently in use to aid smoking cessation and will support future therapeutic developments to treat smoking addiction.

## Methods

### Participating cohorts

#### UK Biobank

The UKB is an open-access, large population cohort of 500,000 individuals established in the United Kingdom^27,63^. The participants were, in general, community-dwelling middle-aged to old-aged volunteers who were recruited between 2006 and 2010 through invitations sent by mail^63^. The age of the participants ranged between 40 and 69 years at the time of recruitment. A deep set of phenotypes has been collected from the participants prospectively, including physical, biochemical and multimodal imaging measures, disease history based on electronic health records (EHRs) and a wide range of environmental measures obtained via touchscreen and web-based questionnaires. The smoking phenotypes that we studied in this project were based on the information collected through lifestyle and environment touchscreen questionnaires (data field category 100058). The health-related phenotypes that we studied including the history of lung and vascular diseases are based on ICD-10 codes from the EHRs or self-reported or a combination of both.

#### Geisinger Health System

The GHS participants come from Geisinger’s MyCode Community Health Initiative, which was established in 2007 to create a biorepository for research projects investigating the molecular and genetic bases of health and disease^50,64^. The participants were patients enrolled in the health care system who consented to participate in the MyCode initiative and gave access to their EHRs. The smoking phenotypes that we studied were based on the clinical history of smoking available in the EHR. Finer details on the smoking behavior such as the number of cigarettes smoked per day, age started smoking, etc. were available for a subset of patients through spirometry questionnaires available in the EHR.

#### Mexico City Prospective Study

The MCPS is a large prospective cohort of 150,000 individuals recruited between 1998 and 2004 with a major aim to investigate the known and new risk factors for mortality in individuals of Mexican descent^46,65^. The participants were residents of the Coyoacan and Iztapalapa districts of Mexico City. Phenotype data including information on smoking behavior were collected through house-to-house visits through interviewer-administered questionnaires.

#### Sinai

The Sinai participants were from the BioMe Biobank Program of the Charles Bronfman Institute for Personalized Medicine at the Mount Sinai Medical Center established in 2007 (ref. ^66^). The BioMe participants are patients enrolled in the Mount Sinai health system who consented to participate in the BioMe initiative and gave access to their EHRs. The smoking phenotypes that we studied were derived from the EHR.

### Ethical approval and informed consent

All study participants have provided informed consent, and all participating cohorts have received ethical approval from their respective institutional review board. The UKB project has received ethical approval from the Northwest Centre for Research Ethics Committee (11/NW/0382)^21,27^. The work described here has been approved by the UKB (application no. 26041)^21^. The GHS project has received ethical approval from the Geisinger Health System Institutional Review Board under project no. 2006-0258 (refs. ^50,64^). The MCPS has received ethical approval from the Mexican Ministry of Health, the Mexican National Council for Science and Technology, the UNAM and the University of Oxford^46,65^. The BioMe biobank has received ethical approval from the institutional review board at the Icahn School of Medicine at Mount Sinai^66^.

### Phenotype definitions

We defined six phenotypes for the primary analysis: (1) ever smoker: cases were those who ever smoked regularly (including both former and current smokers), and controls were those who never smoked in their lifetime; (2) heavy smoker: cases were those who smoked ten or more cigarettes per day (including both former and current smokers), and controls were those who never smoked in their lifetime; (3) former smoker: cases were those who smoked in the past but not at the present, and controls were current smokers; (4) nicotine dependence: cases were those who had an ICD-10 F17 diagnosis in the EHR, and controls were those who did not have an ICD-10 F17 diagnosis; (5) cig per day: number of cigarettes smoked per day in both current and former smokers; (6) age started smoking: age when the person first started smoking.

In addition to the six primary phenotypes, we also studied a set of secondary smoking phenotypes primarily derived from the smoking lifestyle questionnaire data in the UKB (data field category 100058). We also studied a selected list of disease phenotypes related to smoking, namely lung cancer (ICD-10 C34), COPD (ICD-10 J44), emphysema (ICD-10 J43), chronic bronchitis (ICD-10 J42), peripheral arterial disease (ICD-10 I73), coronary artery disease (ICD-10 I25) and myocardial infarction (ICD-10 I21).

### Exome sequencing and variant calling

The exomes of individuals from all participating cohorts were sequenced at the RGC. Exome-sequencing and variant-calling workflows followed for each of the participating cohorts are described in detail elsewhere^10,21,46,64,67^. Briefly, the DNA source for exome sequencing in all the cohorts was peripheral blood. The DNA samples were first enzymatically fragmented into 200-bp DNA libraries, to which 10-bp barcodes were added to facilitate multiplexed operations. Exome regions containing DNA fragments were captured overnight using a modified version of the xGen probe from Integrated DNA Technologies. The captured fragments were then amplified by PCR and sequenced in a multiplexed manner using 75-bp paired-end reads on the Illumina NovaSeq 6000 platform. On average, 20× coverage was achieved for more than 90% of the target sequences in 99% of the samples.

Sequenced reads were mapped to the hg38 reference genome using BWA-MEM to create BAM files. Duplicated reads were marked for exclusion using the Picard tool. Next, variant calling was performed at individual sample levels using the WeCall variant caller to create per-sample gVCF files to enable a sample-level filter. Data from samples with low sequence coverage (<85% of the targeted bases achieving >20× coverage), excess heterozygosity, disagreement between genetic and reported sex, disagreement between exome and array genotype calls and genetic duplicates were removed. The remaining high-quality gVCF files were merged into a single project-level VCF (pVCF) file using the GLnexus joint genotyping tool. A further variant-level filter was applied to the multi-sample pVCF file. SNVs with read depth <7 and indels with read depth <10 were removed. Also, variants without either at least a single homozygous genotype or a single heterozygous genotype with allele balance ratio ≥0.15 (≥0.20 if indel) were removed. The quality-controlled pVCF files were then converted to analysis-ready PGEN format using PLINK version 2.

### Variant annotation

Variants called from exome-sequencing data were annotated using the SnpEff tool^68^. Each variant was assigned the most severe consequence across all the protein-coding transcripts for which start and end positions were defined according to Ensembl release 85. Variants with any of the following annotations: stop gain, start lost, splice donor, splice acceptor, stop lost and frameshift corresponding to the non-ancestral allele were annotated as pLOF variants. Missense deleteriousness was predicted using five different algorithms, namely SIFT^69^, PolyPhen-2 HDIV and PolyPhen-2 HVAR^70^, LRT^71^ and MutationTaster^72^, and missense variants that were predicted to be deleterious by all five algorithms were annotated as ‘likely deleterious’ variants.

### Genotyping and imputation

Genotyping was performed using DNA genotyping arrays that varied from cohort to cohort and are reported in detail in cohort-specific publications^27,46,64^. Briefly, UKB participants were genotyped using the Applied Biosystems UK BiLEVE Axiom Array or the Applied Biosystems UKB Axiom Array; GHS participants were genotyped using either the Illumina Infinium OmniExpressExome or the Global Screening Array; and MCPS and Sinai participants were genotyped using the Global Screening Array. Standard quality-control procedures were followed to retain only high-quality genotyped variants, which were then used for imputing common variants using the TOPMed LD reference panel^73^. For all cohorts, imputation was performed in the TOPMed Imputation Server by uploading the quality-controlled genotypes in randomized batches. Following imputation, we retained only variants with MAF > 0.01 and imputation INFO score > 0.8 for the analysis reported in the current study. After all quality control, the final number of common variants included in the cross-ancestry meta-analyses ranged from ~6.7 million for the ever smoker phenotype to ~14 million variants for the cig-per-day phenotype (the final number of variants decreased as expected with increases in the number of cohorts included in the meta-analyses). Appropriate variables for the genotyping arrays and the imputation batches were used as covariates in all analyses of imputed variants.

### Genetic ancestry inference

Genetic ancestries of the individuals from all participating cohorts were quantified using a set of common variants that were genotyped directly using the genotyping arrays^21^. We first computed principal components (PCs) in HapMap3 individuals using the publicly available genotype reference panel^74^; only high-confidence variants (MAF > 0.10, genotype missingness < 5% and Hardy–Weinberg equilibrium test *P* > 1 × 10^−5^) that were common between our dataset and HapMap3 were used for PC calculations. PCs were first computed in the HapMap3 samples on which the rest of the samples were projected. Individuals were assigned to one of five ancestral groups, namely, Europeans, Africans, AMR, East Asians and South Asians, if their likelihood of belonging to a particular ancestry was >0.3; the likelihood estimate was calculated using a kernel density estimator trained on the HapMap3 PCs^21^.

### Genetic association analysis

Genetic association analyses were performed within each of the cohorts separately using REGENIE software^28^, and the results were then meta-analyzed together using an inverse-variance-weighted approach using METAL software^75^. REGENIE uses a two-step whole-genome regression framework that controls for population stratification and sample relatedness in a cost-effective and computationally efficient manner. Briefly, in step 1, REGENIE computes trait-prediction values (also called local PGS) using a sparse set of genotypes, which are typically the array genotypes. In step 2, REGENIE computes the variant associations with phenotypes using either logistic or linear regression, where the trait-prediction values computed in step 1 are included as covariates along with other covariates, namely the first 20 genetic PCs computed using common variants, the first 20 genetic PCs computed using rare variants, age, age squared, sex, an interaction term between age and sex and genotyping batches. Specifically, for binary traits with imbalanced case–control ratios, REGENIE uses a fast Firth regression, which has been shown to perform better than saddlepoint-approximation correction used in the logistic mixed-model approach implemented in software such as SAIGE^76^. For burden analysis, REGENIE first creates a pseudo-genotype, described as a burden mask, by collapsing a set of variants (see Supplementary Table 2 for the different burden definitions used) into a single categorical variable and then treats this burden mask in the same manner as a variant genotype to compute association statistics.

For the top burden associations, we performed a sensitivity analysis called LOVO implemented in REGENIE. To perform LOVO, REGENIE creates a series of burden masks iteratively for a given set of variants, where, during each iteration, one variant is left out of the burden mask. The created burden masks are then tested for association with the phenotype of interest. Variants that contribute substantially to the burden association will cause a large drop in the statistical significance when left out. Therefore, such an approach can isolate variants that are mainly driving the association and can help evaluate whether a burden association is driven by multiple variants or only a single variant; this is important, as, in the latter, the inferred effect direction cannot be attributed to all variants that were included in the burden mask.

For the top burden associations, we also tested whether the associations were driven by any nearby common variant signals. For this, we iteratively included the most significant common variant observed within 1 Mb on either side of the gene start as a covariate in the REGENIE regression analysis until no nearby common variants with *P* < 0.01 were observed. The burden results from the conditional analysis in each of the cohorts were then meta-analyzed together.

### FinnGen analysis

We downloaded the associations of variant rs202079239 with 3,095 disease endpoints in the FinnGen database using their web browser (https://r7.finngen.fi/variant/1-154575801-C-G)^30^. Through a string search, we extracted associations related to smoking, substance abuse, addiction, COPD and other lung diseases. To test for enrichment of protective associations (OR < 1) in the extracted phenotypes, we did a hypergeometric test using the ‘phyper’ function implemented in the R base package by passing the following values: *q* = 36 (number of associations with OR < 1 among the smoking-related phenotypes), *m* = 2,018 (number of associations with OR < 1 among all phenotypes), *n* = 1,077 (number of associations with OR > 1 among all phenotypes) and *k* = 47 (total number of smoking-related phenotypes extracted).

### Association of rare variant burden at known GWAS loci

The most recent GWAS by Saunders et al. has identified 1,647 loci associated with one or more smoking traits, and, furthermore, the authors have mapped a set of ‘high-priority genes’ through statistical fine mapping^19^. Leveraging these results, we analyzed rare variant burden associations with our six primary smoking phenotypes focused on two gene sets: high-priority genes (*n* genes = 788) and a broader list of genes that are located close to any of the 1,647 GWAS loci reported by Saunders et al. (*n* genes = 1,177)^19^. Similar to our primary analysis, we studied pLOF-only and pLOF-plus-deleterious missense variant burden at five allele frequency thresholds for each of the genes. We applied an FDR of 1% to correct the *P* values for multiple testing.

### CHIP mutation analysis

We identified CHIP mutations in the exome-sequencing data of UKB and GHS participants using a somatic mutation-calling pipeline, which we have described in detail in a previous publication focused on CHIP^33^. Briefly, we used the somatic mutation caller Mutect2, which uses variant mapping and allele-frequency measures to call somatic mutations against a background of germline variants and sequencing errors. CHIP mutation calls were then refined using exome data of a set of reference individuals without somatic mutations (sampled from the lower tail of the age distribution). This was followed by a series of quality-control filtering to identify a final set of highly confident CHIP mutations. In the current work, we studied only the CHIP mutations identified in the eight most recurrent CHIP genes (*DNMT3A*, *TET2*, *ASXL1*, *PPM1D*, *TP53*, *JAK2*, *SRSF2* and *SF3B1*)^33^.

To test whether the ExWAS associations of *ASXL1* and *DNMT3A* are driven by CHIP mutations, we constructed gene burden masks that excluded CHIP mutations and performed burden association tests using REGENIE and compared with the results based on burden masks that included all rare variants. Furthermore, we constructed burden masks for all eight recurrent CHIP genes using only the CHIP mutations and performed burden analysis using REGENIE. We also tested the associations of VAF of the CHIP mutations with the six smoking phenotypes in a merged genetic dataset of CHIP mutation carriers in the UKB (*n* = 28,348) and the GHS (*n* = 11,063) cohorts. We aggregated the VAF estimates for CHIP mutations within each (and across all) of the eight genes and tested their associations with smoking phenotypes through regression analysis adjusted for age, sex, the first ten genetic PCs and a dummy variable for the cohort of origin.

### Identification of independent known and new GWAS loci

To define approximate LD-independent GWAS signals, we used conditional and joint analysis (COJO) implemented in the GCTA software^77^. For the LD reference, we used individual-level genotype data of 10,000 randomly sampled unrelated individuals of either European ancestry (for cross-ancestry and European-specific GWAS) or AMR ancestry (for AMR-specific GWAS). The standard errors of the GWAS summary statistics were adjusted for the LD score regression intercept (LD score regression analysis) before GCTA-COJO analysis. We defined GWAS loci as ‘known’ if the index variant in the loci was in LD (*R*^2^ > 0.1) with genome-wide significant variants reported previously^16^. LD calculations were carried out using PLINK version 2 (ref. ^78^). Our list of known GWAS loci came primarily from Liu et al.^16^. However, before declaring a variant as ‘new’, we also manually queried the variants in the GWAS Catalog to ensure that the variants were not in LD with variants reported in other smoking GWAS publications.

### LD score regression analysis

We calculated SNP-*h*^2^, that is, the proportion of phenotypic variance explained by the common variants, using LD score regression software^43^. We used a European LD reference panel built in house using a random set of 10,000 unrelated European individuals from the UKB following the instructions provided by the authors of the LD score regression software. Genetic correlations were also computed using LD score regression software using the European LD reference panel. We used LD score regression also to quantify the population stratification that is known to inflate GWAS association statistics^43^. We computed LD score intercepts for all GWAS runs including the cross-ancestry and AMR-specific GWAS and then compared the values to the corresponding genomic control (GC) *λ* values. A GC *λ* > 1 but an intercept = 1 suggests that the observed inflation in the test statistics is fully due to polygenicity. For phenotypes such as smoking that are substantially influenced by environmental factors, it is common to have intercept values slightly above 1 (but still lower than GC *λ*), indicating that there is inflation in test statistics due to factors other than polygenicity, for example, population stratification, cryptic relatedness, etc.^43^. To remove such inflation, we applied a correction factor^79^ to the test statistics to constrain the LD score intercept close to 1. We scaled the standard errors of the variant associations by a factor of the square root of the LD score intercept. This is a better alternative to GC correction (commonly practiced in large-scale consortium GWAS), as GC correction tends to overcorrect the statistics, removing true polygenic signals^79^. The LD score statistics before and after intercept correction are reported in Supplementary Table 15. We used the European LD reference panel even for cross-ancestry as well as AMR-specific GWAS, as there are no well-established guidelines on how to handle cross-ancestry or admixed ancestry-based GWAS results. We acknowledge that this has likely biased the results toward variants that are shared between European and other ancestries.

### Polygenic score analysis

We calculated smoking PGS for the UKB participants using SNP weights based on a GWAS of the ever smoker phenotype conducted in an independent sample. We obtained the summary statistics of the most recent GWAS of the ever smoker phenotype from the GSCAN consortium based on an analysis of all the participating GSCAN cohorts except the UKB and 23andMe^19^. To improve the statistical power of the PGS, we meta-analyzed the GSCAN results with the GWAS results of the GHS cohort, which together yielded a total sample size of 482,096 individuals. We then refined the SNP effect sizes in the GWAS summary statistics using PRS-CS software^80^, which uses a Bayesian approach to calculate SNP posterior effect sizes under continuous shrinkage priors based on an external LD reference panel. The refined SNP weights are then used to compute PGS using PLINK version 2 software^78^.

We performed two types of analysis. First, we studied the associations of PGS and *CHRNB2* pLOF-plus-missense burden with heavy smoking using logistic regression analysis, in which the heavy smoker phenotype was coded as the dependent variable (that is, outcome), and PGS, burden mask, an interaction term between PGS and burden mask and relevant covariates (the same as the ones used in the GWAS) were coded as independent variables (regression formula: heavy smoker ≈ PGS + burden mask + PGS × burden mask + covariate_1_ + …covariate_*n*_). Second, we binned UKB individuals into quintiles (five equally sized groups) based on their smoking PGS. Individuals within each quintile were further divided into carriers and non-carriers of *CHRNB2* pLOF or likely deleterious missense variants at MAF < 0.001. The prevalence of heavy smokers was then compared between carriers and non-carriers within each quintile; the standard error was calculated using the formula √(*pq*/*n*), where *n* is the number of individuals in the group, *p* is the prevalence of heavy smokers in the group and *q* is 1 − *p*. We also tested the statistical difference in the prevalence of heavy smokers between carriers and non-carriers of rare variant burden using logistic regression analysis adjusted for relevant covariates (the same as the ones used in the GWAS). The OR, 95% CI and the *P* value for each quintile are reported in Fig. 7.

### Power calculations

All power calculations were carried out in R using the package ‘genpwr’ available from CRAN^81^. In all cases, we computed effect sizes (*β* values) using the function ‘genpwr.calc’ with the following input parameters: power = 0.80, calc = ‘es’, model = ‘logistic’ for binary phenotypes and ‘linear’ for quantitative phenotypes, *α* = ‘5 × 10^−8^’ for GWAS and ‘4.5 × 10^−8^’ for ExWAS, MAF = values ranging from 0 to 0.5, True.model = ‘additive’ and Test.model = ‘additive’, *n* = total sample size, case_rate = *n* cases(*n* total)^−1^ (for binary phenotypes) and sd_y = 1 (for quantitative phenotypes).

### Reporting summary

Further information on research design is available in the Nature Portfolio Reporting Summary linked to this article.

## Online content

Any methods, additional references, Nature Portfolio reporting summaries, source data, extended data, supplementary information, acknowledgements, peer review information; details of author contributions and competing interests; and statements of data and code availability are available at 10.1038/s41588-023-01417-8.

## Supplementary information

Supplementary Information Supplementary Figs. 1–6 and Note.

Reporting Summary

Peer Review File

Supplementary Tables Supplementary Tables 1–17.

## Data Availability

The data supporting the findings of this study are reported in the main text, figures and Supplementary Tables 1–17. UKB individual-level genotypic and phenotypic data are available to approved investigators via the UKB study (https://www.ukbiobank.ac.uk/). Additional information about registration for access to the data is available at https://www.ukbiobank.ac.uk/register-apply/. Data access for approved applications requires a data-transfer agreement between the researcher’s institution and the UKB, the terms of which are available on the UKB website (https://www.ukbiobank.ac.uk/media/ezrderzw/applicant-mta.pdf). GHS individual-level data are available to qualified academic noncommercial researchers through the portal at https://regeneron.envisionpharma.com/vt_regeneron/ under a data-access agreement. The MCPS represents a long-standing collaboration between researchers at the UNAM and the University of Oxford. The investigators welcome requests from researchers in Mexico and elsewhere who wish to access MCPS data. If you are interested in obtaining data from the study for research purposes or in collaborating with MCPS investigators on a specific research proposal, please visit https://www.ctsu.ox.ac.uk/research/mcps, where you can download the study’s Data and Sample Access Policy in English or Spanish. The policy lists the data available for sharing with researchers in Mexico and in other parts of the world. Full details of the available data may also be viewed at https://datashare.ndph.ox.ac.uk/. FinnGen release 7 genetic association results, which were used in the current study, are publicly available at https://r7.finngen.fi/.

## Code availability

All genetic association analyses were performed using REGENIE software version 2.0.1, developed in house. REGENIE software is freely available on GitHub (https://github.com/rgcgithub/regenie) and Zenodo (10.5281/zenodo.6789126)^82^.

## References

[1] Reitsma MB (2021). Spatial, temporal, and demographic patterns in prevalence of smoking tobacco use and attributable disease burden in 204 countries and territories, 1990–2019: a systematic analysis from the Global Burden of Disease Study 2019. Lancet.

[2] Rigotti NA, Kruse GR, Livingstone-Banks J, Hartmann-Boyce J (2022). Treatment of tobacco smoking: a review. JAMA.

[3] Jordan CJ, Xi Z-X (2018). Discovery and development of varenicline for smoking cessation. Expert Opin. Drug Discov..

[4] Tong EK, Carmody TP, Simon JA (2006). Bupropion for smoking cessation: a review. Compr. Ther..

[5] US Preventive Services Task Force (2021). Interventions for tobacco smoking cessation in adults, including pregnant persons: US Preventive Services Task Force Recommendation Statement. JAMA.

[6] Brown KM (2022). Expanding RNAi therapeutics to extrahepatic tissues with lipophilic conjugates. Nat. Biotechnol..

[7] Szustakowski JD (2021). Advancing human genetics research and drug discovery through exome sequencing of the UK Biobank. Nat. Genet..

[8] Stitziel NO, Kathiresan S (2017). Leveraging human genetics to guide drug target discovery. Trends Cardiovasc. Med..

[9] Verweij N (2022). Germline mutations in *CIDEB* and protection against liver disease. N. Engl. J. Med..

[10] Akbari P (2021). Sequencing of 640,000 exomes identifies *GPR75* variants associated with protection from obesity. Science.

[11] Minicocci I (2012). Mutations in the *ANGPTL3* gene and familial combined hypolipidemia: a clinical and biochemical characterization. J. Clin. Endocrinol. Metab..

[12] Cohen JC, Boerwinkle E, Mosley TH, Hobbs HH (2006). Sequence variations in *PCSK9*, low LDL, and protection against coronary heart disease. N. Engl. J. Med..

[13] Robinson JG (2015). Efficacy and safety of alirocumab in reducing lipids and cardiovascular events. N. Engl. J. Med..

[14] Vink JM, Willemsen G, Boomsma DI (2005). Heritability of smoking initiation and nicotine dependence. Behav. Genet..

[15] Jang S-K (2022). Rare genetic variants explain missing heritability in smoking. Nat. Hum. Behav..

[16] Liu M (2019). Association studies of up to 1.2 million individuals yield new insights into the genetic etiology of tobacco and alcohol use. Nat. Genet..

[17] Xu K (2020). Genome-wide association study of smoking trajectory and meta-analysis of smoking status in 842,000 individuals. Nat. Commun..

[18] Erzurumluoglu, A. M. (2020). Meta-analysis of up to 622,409 individuals identifies 40 novel smoking behaviour associated genetic loci. Mol. Psychiatry.

[19] Saunders GRB (2022). Genetic diversity fuels gene discovery for tobacco and alcohol use. Nature.

[20] Brazel DM (2019). Exome chip meta-analysis fine maps causal variants and elucidates the genetic architecture of rare coding variants in smoking and alcohol use. Biol. Psychiatry.

[21] Backman JD (2021). Exome sequencing and analysis of 454,787 UK Biobank participants. Nature.

[22] Smelser DT (2022). Association of varicose veins with rare protein-truncating variants in *PIEZO1* identified by exome sequencing of a large clinical population. J. Vasc. Surg. Venous Lymphat. Disord..

[23] Ruth KS (2021). Genetic insights into biological mechanisms governing human ovarian ageing. Nature.

[24] Raal FJ (2020). Evinacumab for homozygous familial hypercholesterolemia. N. Engl. J. Med..

[25] McGregor TL (2020). Characterising a healthy adult with a rare *HAO1* knockout to support a therapeutic strategy for primary hyperoxaluria. eLife.

[26] A phase 1/2, randomized, double-blind, placebo-controlled, single ascending and multiple dose study of the safety, tolerability, efficacy, pharmacokinetics, and pharmacodynamics of ALN-XDH in healthy adult subjects and adult patients with gout. https://clinicaltrials.gov/ct2/show/NCT05256810 (2022).

[27] Bycroft C (2018). The UK Biobank resource with deep phenotyping and genomic data. Nature.

[28] Mbatchou J (2021). Computationally efficient whole-genome regression for quantitative and binary traits. Nat. Genet..

[29] Karczewski KJ (2020). The mutational constraint spectrum quantified from variation in 141,456 humans. Nature.

[30] Kurki MI (2023). FinnGen provides genetic insights from a well-phenotyped isolated population. Nature.

[31] Asada S, Kitamura T (2021). Clonal hematopoiesis and associated diseases: a review of recent findings. Cancer Sci..

[32] Kar SP (2022). Genome-wide analyses of 200,453 individuals yield new insights into the causes and consequences of clonal hematopoiesis. Nat. Genet..

[33] Kessler MD (2022). Common and rare variant associations with clonal haematopoiesis phenotypes. Nature.

[34] Zink F (2017). Clonal hematopoiesis, with and without candidate driver mutations, is common in the elderly. Blood.

[35] Dawoud AAZ, Tapper WJ, Cross NCP (2020). Clonal myelopoiesis in the UK Biobank cohort: *ASXL1* mutations are strongly associated with smoking. Leukemia.

[36] Carlston CM (2017). Pathogenic *ASXL1* somatic variants in reference databases complicate germline variant interpretation for Bohring–Opitz syndrome. Hum. Mutat..

[37] Brunet T (2022). Clonal hematopoiesis as a pitfall in germline variant interpretation in the context of Mendelian disorders. Hum. Mol. Genet..

[38] Chanock SJ, Hunter DJ (2008). When the smoke clears. Nature.

[39] Berrettini WH, Doyle GA (2012). The *CHRNA5*–*A3*–*B4* gene cluster in nicotine addiction. Mol. Psychiatry.

[40] Thorgeirsson, T. E. (2010). Sequence variants at *CHRNB3*–*CHRNA6* and *CYP2A6* affect smoking behavior. Nat. Genet..

[41] Amos CI, Spitz MR, Cinciripini P (2010). Chipping away at the genetics of smoking behavior. Nat. Genet..

[42] Kim YJ (2022). The contribution of common and rare genetic variants to variation in metabolic traits in 288,137 East Asians. Nat. Commun..

[43] Bulik-Sullivan, B. K. (2015). LD Score regression distinguishes confounding from polygenicity in genome-wide association studies. Nat. Genet..

[44] Khasabov SG (2021). The nAChR chaperone TMEM35a (NACHO) contributes to the development of hyperalgesia in mice. Neuroscience.

[45] Ochoa D (2021). Open Targets Platform: supporting systematic drug–target identification and prioritisation. Nucleic Acids Res..

[46] Ziyatdinov, A. et al. Genotyping, sequencing and analysis of 140,000 adults from the Mexico City Prospective Study. Preprint at *bioRxiv*10.1101/2022.06.26.495014 (2022).

[47] Saccone NL (2018). Genome-wide association study of heavy smoking and daily/nondaily smoking in the Hispanic Community Health Study/Study of Latinos (HCHS/SOL). Nicotine Tob. Res..

[48] Hancock DB (2015). Genome-wide meta-analysis reveals common splice site acceptor variant in *CHRNA4* associated with nicotine dependence. Transl. Psychiatry.

[49] Song W, Lin GN, Yu S, Zhao M (2022). Genome-wide identification of the shared genetic basis of cannabis and cigarette smoking and schizophrenia implicates *NCAM1* and neuronal abnormality. Psychiatry Res..

[50] Carey DJ (2016). The Geisinger MyCode Community Health Initiative: an electronic health record-linked biobank for precision medicine research. Genet. Med..

[51] Zeggini E (2014). Using genetically isolated populations to understand the genomic basis of disease. Genome Med..

[52] Koch L (2021). The power of large-scale exome sequencing. Nat. Rev. Genet..

[53] Wong DF (2013). PET imaging of high-affinity α4β2 nicotinic acetylcholine receptors in humans with ^18^F-AZAN, a radioligand with optimal brain kinetics. J. Nucl. Med..

[54] Picciotto MR, Kenny PJ (2021). Mechanisms of nicotine addiction. Cold Spring Harb. Perspect. Med..

[55] Marks MJ (2013). Genetic matters: thirty years of progress using mouse models in nicotinic research. Biochem. Pharmacol..

[56] Picciotto MR (1995). Abnormal avoidance learning in mice lacking functional high-affinity nicotine receptor in the brain. Nature.

[57] Picciotto MR (1998). Acetylcholine receptors containing the β2 subunit are involved in the reinforcing properties of nicotine. Nature.

[58] Karnieg T, Wang X (2018). Cytisine for smoking cessation. CMAJ.

[59] Bagdas D (2018). New insights on the effects of varenicline on nicotine reward, withdrawal and hyperalgesia in mice. Neuropharmacology.

[60] Ju D, Hui D, Hammond DA, Wonkam A, Tishkoff SA (2022). Importance of including non-European populations in large human genetic studies to enhance precision medicine. Annu. Rev. Biomed. Data Sci..

[61] Sirugo G, Williams SM, Tishkoff SA (2019). The missing diversity in human genetic studies. Cell.

[62] Halldorsson BV (2022). The sequences of 150,119 genomes in the UK Biobank. Nature.

[63] Sudlow C (2015). UK Biobank: an open access resource for identifying the causes of a wide range of complex diseases of middle and old age. PLoS Med..

[64] Dewey FE (2016). Distribution and clinical impact of functional variants in 50,726 whole-exome sequences from the DiscovEHR study. Science.

[65] Tapia-Conyer R (2006). Cohort profile: the Mexico City Prospective Study. Int. J. Epidemiol..

[66] Belbin GM (2021). Leveraging health systems data to characterize a large effect variant conferring risk for liver disease in Puerto Ricans. Am. J. Hum. Genet..

[67] Van Hout CV (2020). Exome sequencing and characterization of 49,960 individuals in the UK Biobank. Nature.

[68] Cingolani P (2012). A program for annotating and predicting the effects of single nucleotide polymorphisms, SnpEff. Fly.

[69] Vaser R, Adusumalli S, Leng SN, Sikic M, Ng PC (2016). SIFT missense predictions for genomes. Nat. Protoc..

[70] Adzhubei I, Jordan DM, Sunyaev SR (2013). Predicting functional effect of human missense mutations using PolyPhen-2. Curr. Protoc. Hum. Genet..

[71] Chun S, Fay JC (2009). Identification of deleterious mutations within three human genomes. Genome Res..

[72] Schwarz JM, Rödelsperger C, Schuelke M, Seelow D (2010). MutationTaster evaluates disease-causing potential of sequence alterations. Nat. Methods.

[73] Taliun, D. (2021). Sequencing of 53,831 diverse genomes from the NHLBI TOPMed Program. Nature.

[74] The International HapMap 3 Consortium. (2010). Integrating common and rare genetic variation in diverse human populations. Nature.

[75] Willer CJ, Li Y, Abecasis GR (2010). METAL: fast and efficient meta-analysis of genomewide association scans. Bioinformatics.

[76] Zhou W (2018). Efficiently controlling for case–control imbalance and sample relatedness in large-scale genetic association studies. Nat. Genet..

[77] Yang J, Lee SH, Goddard ME, Visscher PM (2011). GCTA: a tool for genome-wide complex trait analysis. Am. J. Hum. Genet..

[78] Chang CC (2015). Second-generation PLINK: rising to the challenge of larger and richer datasets. GigaScience.

[79] Yengo L (2018). Meta-analysis of genome-wide association studies for height and body mass index in ∼700000 individuals of European ancestry. Hum. Mol. Genet..

[80] Ge T, Chen C-Y, Ni Y, Feng Y-CA, Smoller JW (2019). Polygenic prediction via Bayesian regression and continuous shrinkage priors. Nat. Commun..

[81] Moore CM, Jacobson SA, Fingerlin TE (2019). Power and sample size calculations for genetic association studies in the presence of genetic model misspecification. Hum. Hered..

[82] Mbatchou, J. et al. rgcgithub/regenie: Regenie v3.2.6. *Zenodo*10.5281/zenodo.7838822 (2023).

