# Supplementary material for: Rare coding variants in CHRNB2 reduce the likelihood of smoking
Source: Nat Genet. 2023 Jun 12;55(7):1138–48. doi: 10.1038/s41588-023-01417-8 (PMC10335934; doi:10.1038/s41588-023-01417-8)
Supplement: Supplementary file 1 — Supplementary Figs. 1–6 and Note. [file 41588_2023_1417_MOESM1_ESM.pdf]

nature genetics

Article https://doi.org/10.1038/s41588-023-01417-8

Rare coding variants in *CHRNB2* reduce the likelihood of smoking

In the format provided by the authors and unedited

Table of Contents

Supplementary Figures ***2***

Supplementary Figure 1 .... **2**

Supplementary Figure 2 .... **5**

Supplementary Figure 3 .... **6**

Supplementary Figure 4 .... **6**

Supplementary Figure 5 .... **7**

Supplementary Figure 6 .... **9**

Supplementary Notes ***12***

Association of CHIP genes with smoking phenotypes **12**

CHIP mutations are pathogenic when occurring in the germline. **12**

References ***12***

Supplementary Figures

Supplementary Figure 1

a.

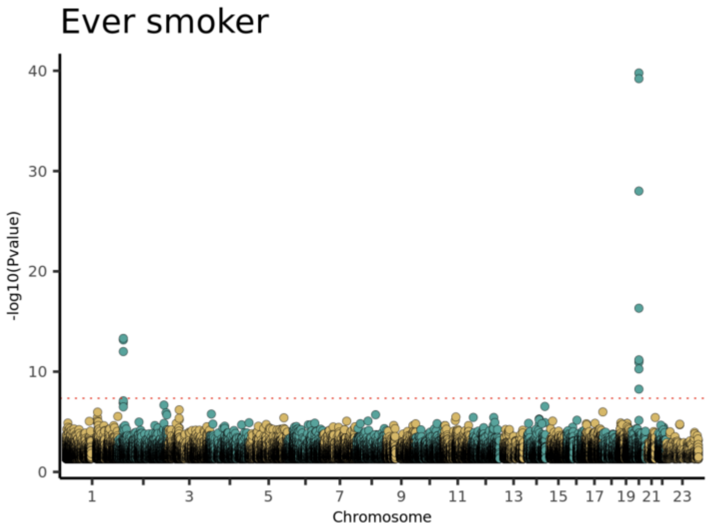

b.

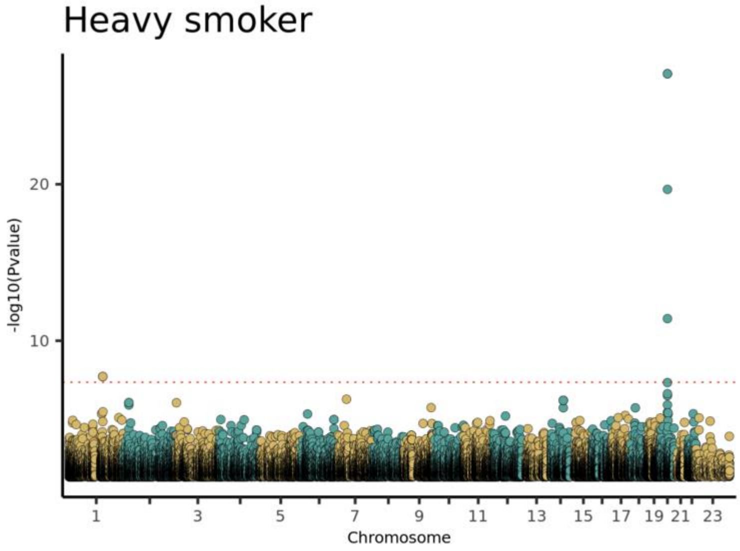

c.

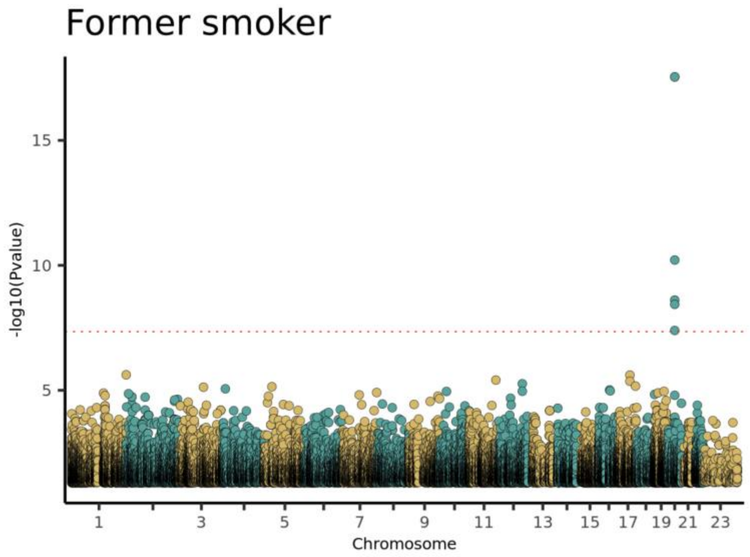
Former smoker
-log10(Pvalue)
15
10
5
Chromosome
1
3
5
7
9
11
13
15
17
19
21
23

d.

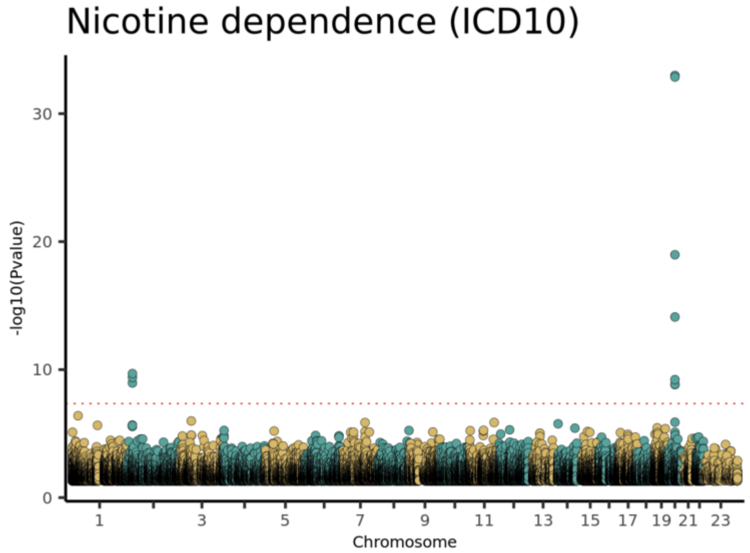
Nicotine dependence (ICD10)
-log10(Pvalue)
30
20
10
0
Chromosome
1
3
5
7
9
11
13
15
17
19
21
23

e.

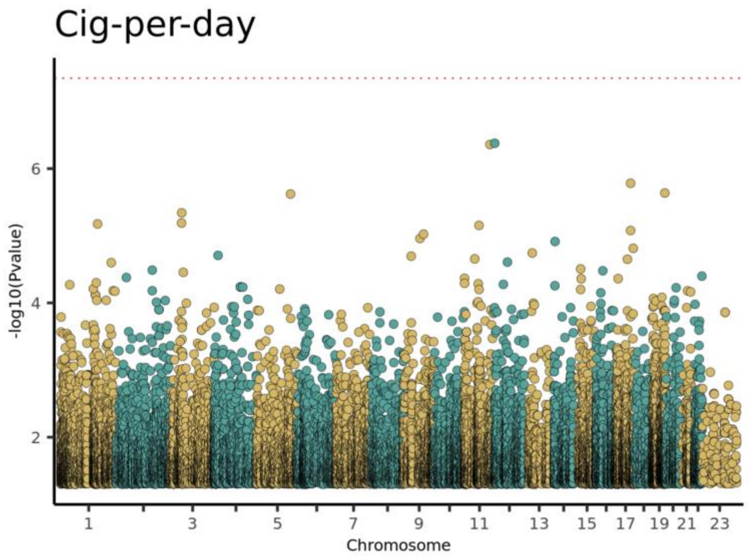

f.

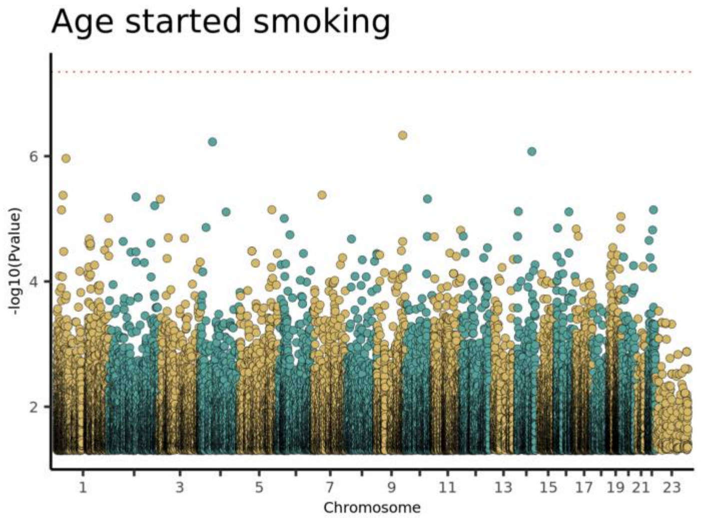

Supplementary Figure 1. ExWAS Manhattan plots of six smoking phenotypes. The plots display genome-wide genetic associations of both individual variants (loss of function and missense variants with MAF<0.01) and gene burden masks (pLOF only and pLOF plus likely deleterious missense variants at five MAF cut-offs: <0.01, <0.001, <0.0001, <0.00001 and Singletons) with six primary smoking phenotypes tested using REGENIE (Methods) (**a-f**). The exome-wide significance P value threshold was calculated by applying a false detection rate (FDR) of 1% across the associations of all six smoking phenotypes (8,417,987 association tests in total), which corresponds to P=4.5e-8, marked with a dotted red line in each of the plots.

Supplementary Figure 2

a.

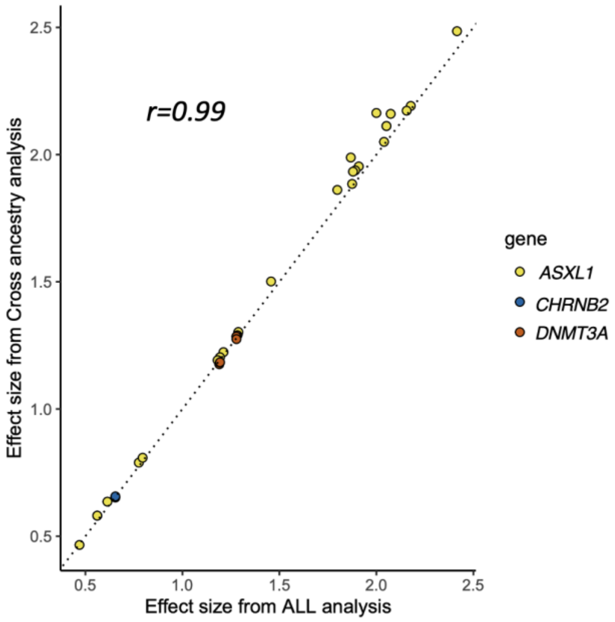

b.

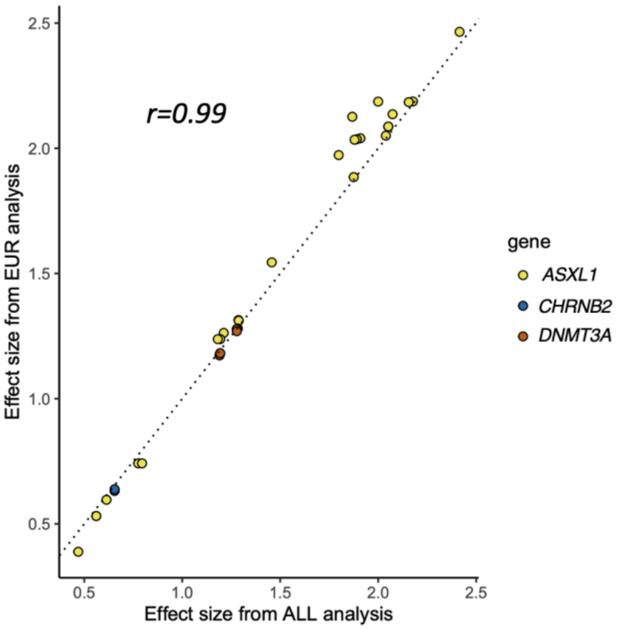

Supplementary Figure 2. Effect size (odds ratio) comparison of 35 significant ExWAS associations a. ALL (all ancestries pooled) meta-analysis vs cross ancestry meta-analysis (ancestry specific analysis followed by meta-analysis) and **b.** ALL meta-analysis vs EUR only meta-analysis. Pearson correlation (r) estimates are shown.

Supplementary Figure 3

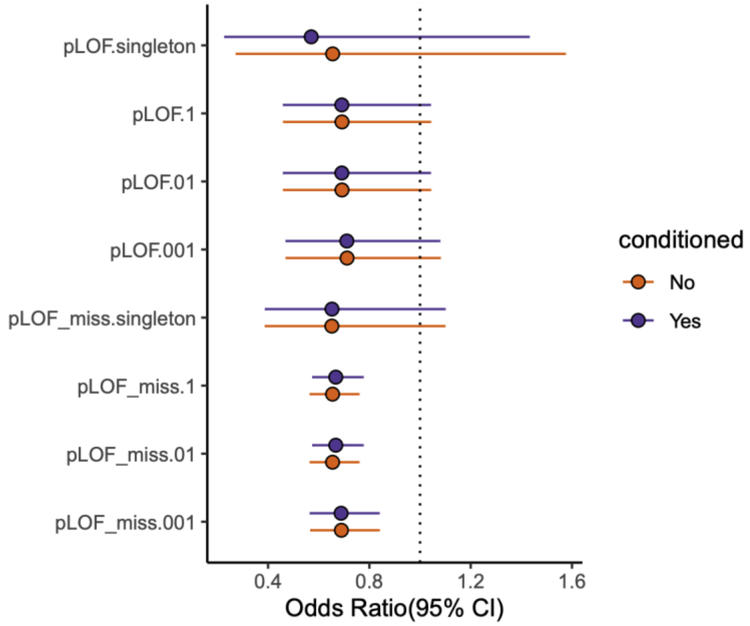

Supplementary Figure 3. Analysis of *CHRNB2* burden associations with *heavy-smoker* conditioned on nearby common variants. *CHRNB2* burden associations with *heavy-smoker* (N cases = 110,494, N controls = 378,842) were computed before and after conditioning on nearby common variants recursively until no variants with 1 Mb on either side of the transcription start site of *CHRNB2* had P<0.01. The odds ratio and 95% confidence intervals from the burden associations before and after conditioning are plotted. The effect sizes remained the same after conditioning on the nearby common variants suggesting the burden associations are independent of any nearby common variant signal.

Supplementary Figure 4

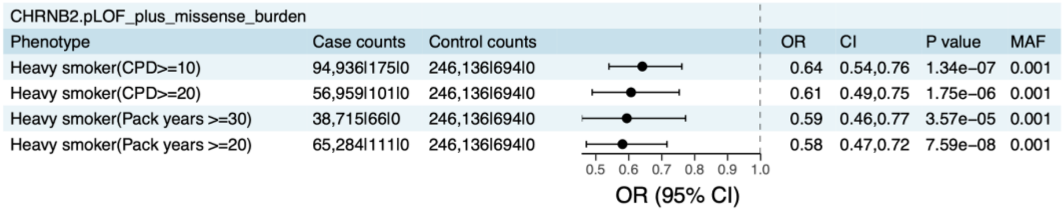

Supplementary Figure 4. Forest plots of associations of *CHRNB2* burden masks with various definitions of heavy-smoker. We defined heavy smoking in the UK Biobank in four different ways: cig-per-day ≥ 10 (the main definition), cig-per-day ≥ 20, smoking pack years ≥ 20 and smoking pack years ≥ 30 and performed rare variant burden analysis for each of the definitions using REGENIE. The odds ratios and 95% confidence intervals of the *CHRNB2* pLOF-plus-missense burden (MAF<0.001) associations with the four definitions of heavy-smoker are shown in the forest plot. The columns 'Case counts' and 'Control counts' provide sample sizes for cases and controls respectively broken down into the number of carriers of wild-type, heterozygous and homozygous genotypes.

Supplementary Figure 5

a.

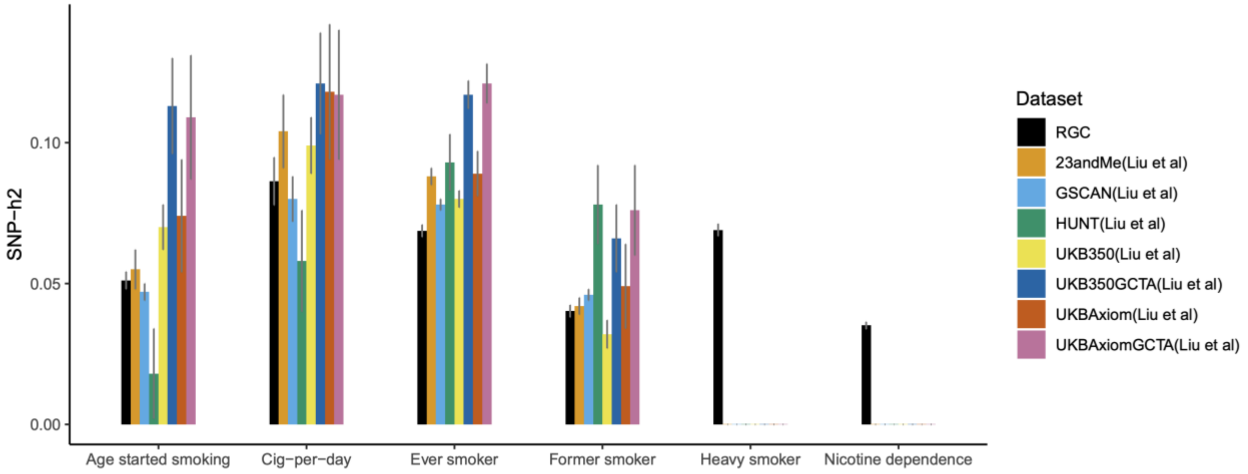

b.

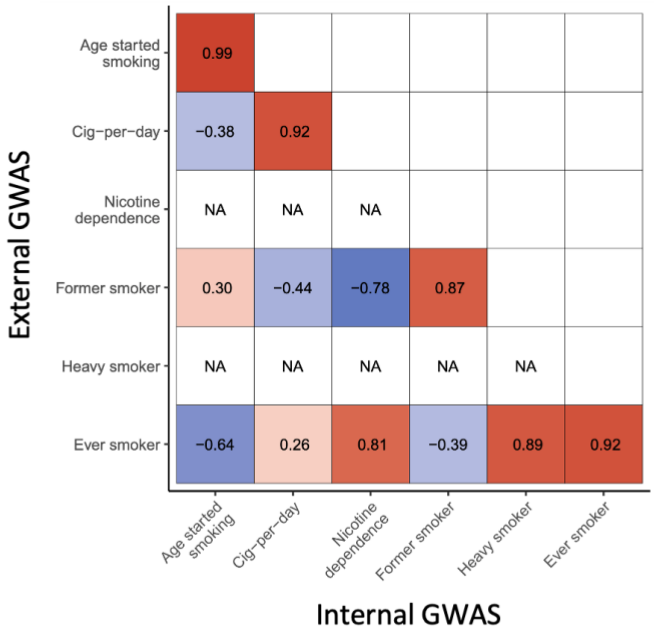

c.

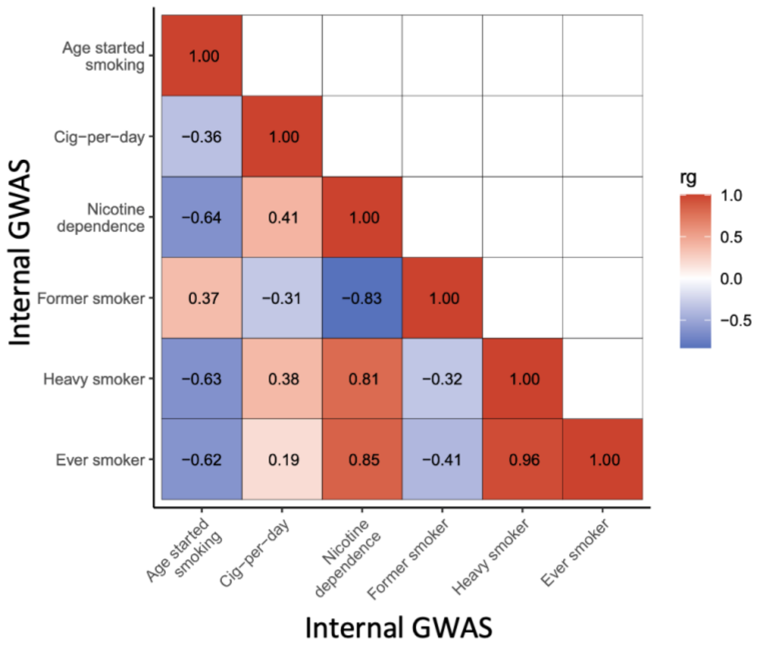

Supplementary Figure 5. SNP-h2 and genetic correlations. **a.** SNP-h2 of the six smoking phenotypes, estimated using LD score regression, are plotted along with standard errors. The SNP-h2 estimates reported by Liu et al 2019 are also plotted for comparison. Note, Liu et al did not study heavy smoker and nicotine dependence phenotypes. **b.** Genetic correlations between our six smoking phenotypes and the four smoking phenotypes from the GSCAN consortium (Liu et al 2019) estimated using LD score regression are shown as heat maps. The rg estimates are displayed over the plots. Nicotine dependence and heavy smoker phenotypes are not studied by Liu et al, hence, shown as NA. **c.** Genetic correlations between the smoking phenotypes within our cohort are displayed.
Internal GWAS – Cohorts involved in the current study
External GWAS – GSCAN cohorts (excluding UKB)

Supplementary Figure 6

a.

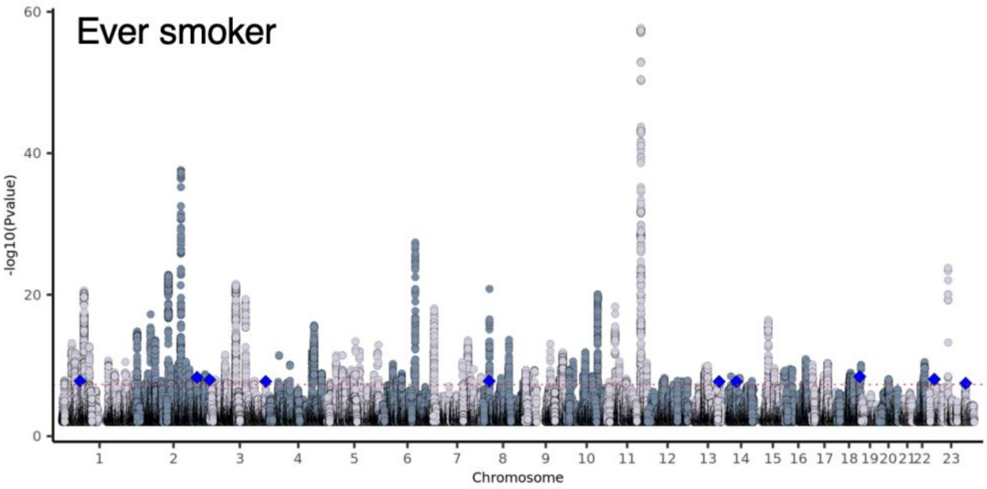

b.

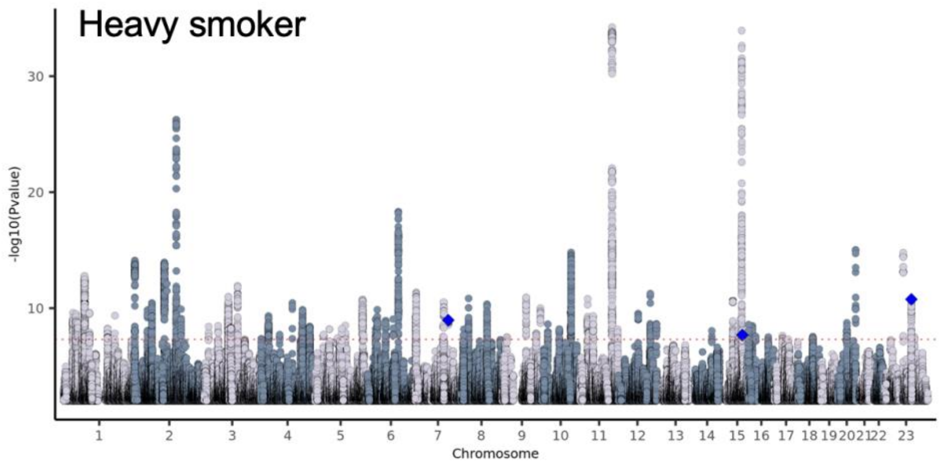

c.

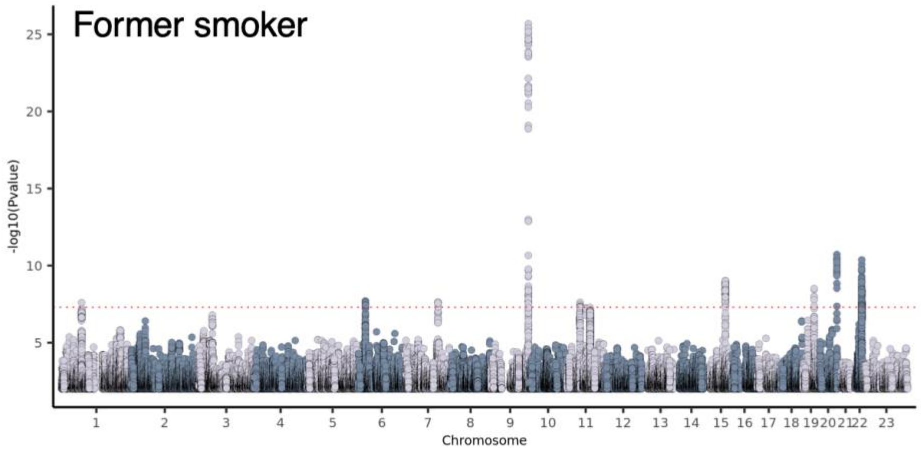
Former smoker
-log10(Pvalue)
25
20
15
10
5
1 2 3 4 5 6 7 8 9 10 11 12 13 14 15 16 17 18 19 20 21 22 23
Chromosome

d.

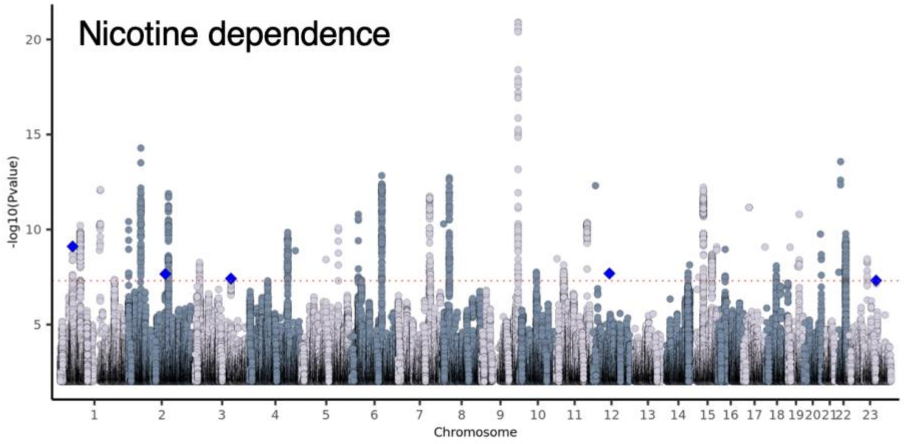
Nicotine dependence
-log10(Pvalue)
20
15
10
5
1 2 3 4 5 6 7 8 9 10 11 12 13 14 15 16 17 18 19 20 21 22 23
Chromosome

e.

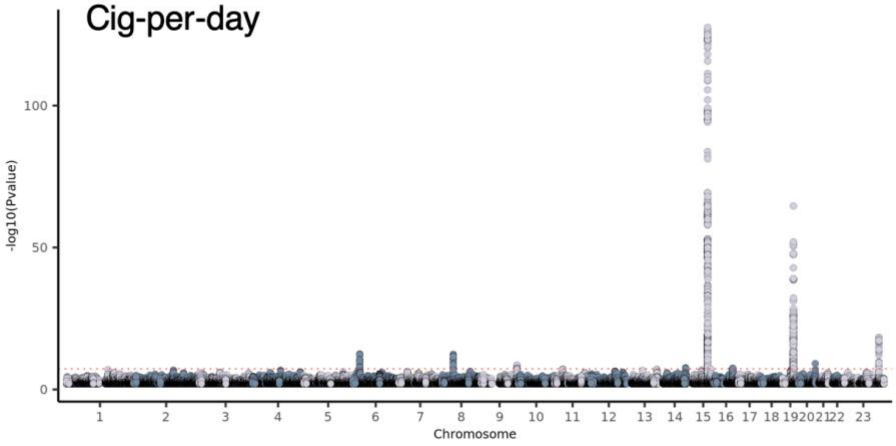
Cig-per-day
-log10(Pvalue)
100
50
0
1 2 3 4 5 6 7 8 9 10 11 12 13 14 15 16 17 18 19 20 21 22 23
Chromosome

f.

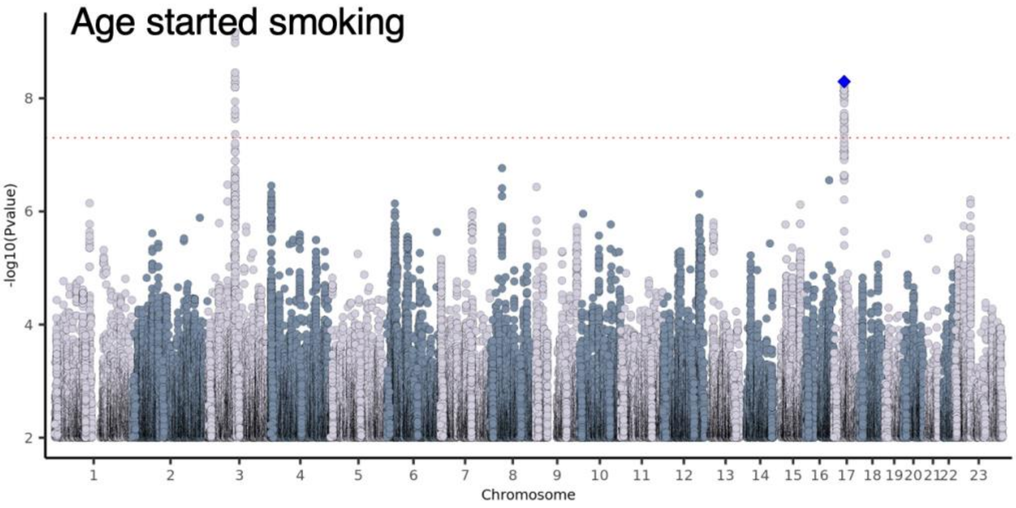

Supplementary Figure 6. Manhattan plots of cross-ancestry GWAS meta-analyses of the six smoking phenotypes (a-f). The novel GWAS loci are marked with blue diamonds in the Manhattan plots. The red dotted line corresponds to P value of 5e-8.

Supplementary Notes

Association of CHIP genes with smoking phenotypes

We observed that *ASXL1* and *DNMT3A* rare variant burden were strongly associated with multiple smoking phenotypes. Given that *ASXL1* and *DNMT3A* are known CHIP genes, we constructed burden masks in the UKB and GHS cohorts after excluding variants that we previously identified as high-confident CHIP mutations[1]. When the associations of *ASXL1* and *DNMT3A* with smoking phenotypes were analyzed using gene burden masks that excluded CHIP mutations, we found associations for neither of the genes with any of the six smoking phenotypes (Fig. 6 and Supplementary Table 8). We further tested the associations of all the eight most recurrently mutated CHIP genes[1] (*DNMT3A, TET2, ASXL1, PPM1D, TP53, SRSF2, JAK2, SF3B1*) with our six smoking phenotypes in the GHS and UKB, using burden masks created using only CHIP mutations (Extended Data Fig. 5a and Supplementary Table 9). In line with our earlier findings, the strongest associations were seen for *ASXL1 and DNMT3A*. In addition, we also observed a significant association (P<0.003 based on 1% FDR across eight genes) for PPM1D pLOF-only burden with heavy-smoker (OR=1.8; CI=1.4-2.3; P=5.5e-7). We also tested the associations of variant allele fractions (VAF) of CHIP mutations aggregated within each (and across all) of the eight recurrent CHIP genes with the six smoking phenotypes (Extended Data Fig. 5c; Methods). After correcting for multiple testing (FDR 1%), the VAF estimates aggregated across all the CHIP genes showed significant associations with *ever-smoker* (beta=0.004; SE=0.001; P=4.4e-5) and heavy-smoker (beta=0.005; SE=0.001; P=1.1e-4). At the level of individual genes, the strongest associations were seen for the VAF of *ASXL1* CHIP mutations with *ever-smoker* and *heavy-smoker* (Extended Data Fig. 5c; Supplementary Table 10).

CHIP mutations are pathogenic when occurring in the germline.

The strongest individual CHIP mutation associated with smoking in our analysis was a frame-shift mutation, rs750318549 (p.Gly646fs), in *ASXL1* (nicotine dependence: OR=2.41; CI=1.9-3.0; P=7.6e-15), which is pathogenic when it occurs in the germline (mostly as de novo), causing a neurodevelopmental disorder called Bohring-Opitz syndrome[2] (Supplementary Table 4). We found 371 heterozygous carriers in the UKB, who were on average 6.3 yrs. (SE=0.4; P=3.5e-51) older than the non-carriers (average age in carriers=62.9±5.56; non-carriers=56.5±8.1). Observing 371 carriers of a pathogenic variant in a cohort of middle to old-aged healthy volunteers strongly points to the somatic origin of this variant.

References

1. Kessler, M. D. *et al.* Common and rare variant associations with clonal haematopoiesis phenotypes. *Nature* **612**, 301–309 (2022).

2. Carlston, C. M. *et al.* Pathogenic *ASXL1* somatic variants in reference databases complicate germline variant interpretation for Bohring-Opitz Syndrome: CARLSTON et al. *Hum. Mutat.* **38**, 517–523 (2017).
