# Supplementary material for: Rare coding variants in CHRNB2 reduce the likelihood of smoking
Source: Nat Genet. 2023 Jun 12;55(7):1138–48. doi: 10.1038/s41588-023-01417-8 (PMC10335934; doi:10.1038/s41588-023-01417-8)
Supplement: Supplementary file 2 — Reporting Summary [file 41588_2023_1417_MOESM2_ESM.pdf]

nature portfolio

Corresponding author(s): Giovanni Coppola, Aris Baras

Last updated by author(s): Apr 28, 2023

Reporting Summary

Nature Portfolio wishes to improve the reproducibility of the work that we publish. This form provides structure for consistency and transparency in reporting. For further information on Nature Portfolio policies, see our Editorial Policies and the Editorial Policy Checklist.

Statistics

For all statistical analyses, confirm that the following items are present in the figure legend, table legend, main text, or Methods section.

n/a	Confirmed	
☐	☒	The exact sample size (*n*) for each experimental group/condition, given as a discrete number and unit of measurement
☐	☒	A statement on whether measurements were taken from distinct samples or whether the same sample was measured repeatedly
☐	☒	The statistical test(s) used AND whether they are one- or two-sided<br>*Only common tests should be described solely by name; describe more complex techniques in the Methods section.*
☐	☒	A description of all covariates tested
☐	☒	A description of any assumptions or corrections, such as tests of normality and adjustment for multiple comparisons
☐	☒	A full description of the statistical parameters including central tendency (e.g. means) or other basic estimates (e.g. regression coefficient) AND variation (e.g. standard deviation) or associated estimates of uncertainty (e.g. confidence intervals)
☐	☒	For null hypothesis testing, the test statistic (e.g. *F*, *t*, *r*) with confidence intervals, effect sizes, degrees of freedom and *P* value noted<br>*Give P values as exact values whenever suitable.*
☒	☐	For Bayesian analysis, information on the choice of priors and Markov chain Monte Carlo settings
☒	☐	For hierarchical and complex designs, identification of the appropriate level for tests and full reporting of outcomes
☐	☒	Estimates of effect sizes (e.g. Cohen's *d*, Pearson's *r*), indicating how they were calculated

Our web collection on statistics for biologists contains articles on many of the points above.

Software and code

Policy information about availability of computer code

Data collection	None
Data analysis	Software used for data analysis include Regenie (v.3.2.1), LDSC(v1.0.1), PRS-CS(v1.0.0), R (v4.1.0), GCTA (v1.91.7), SHAPEIT (v4.2.0), IMPUTE (v5), Mutect2 (GATK v4.1.4.0)

For manuscripts utilizing custom algorithms or software that are central to the research but not yet described in published literature, software must be made available to editors and reviewers. We strongly encourage code deposition in a community repository (e.g. GitHub). See the Nature Portfolio guidelines for submitting code & software for further information.

Data

Policy information about availability of data

All manuscripts must include a data availability statement. This statement should provide the following information, where applicable:

- Accession codes, unique identifiers, or web links for publicly available datasets
- A description of any restrictions on data availability
- For clinical datasets or third party data, please ensure that the statement adheres to our policy

UKB individual-level genotypic and phenotypic data are available to approved investigators via the UK Biobank study (www.ukbiobank.ac.uk/). Additional information about registration for access to the data are available at www.ukbiobank.ac.uk/register-apply/. Data access for approved applications requires a data transfer agreement between the researcher's institution and UK Biobank, the terms of which are available on the UK Biobank website (www.ukbiobank.ac.uk/

media/ezrderzw/applicant-mta.pdf). GHS individual-level data are available to qualified academic noncommercial researchers through the portal at https://regeneron.envisionpharma.com/vt_regeneron/ under a data access agreement. The MCPS represents a long-standing collaboration between researchers at the National Autonomous University of Mexico (UNAM) and the University of Oxford. The investigators welcome requests from researchers in Mexico and elsewhere who wish to access MCPS data. If you are interested in obtaining data from the study for research purposes, or in collaborating with MCPS investigators on a specific research proposal, please visit https://www.ctsu.ox.ac.uk/research/prospective-blood-based-study-of-150-000-individuals-in-mexico where you can download the study's Data and Sample Access Policy in English or Spanish. The policy lists the data available for sharing with researchers in Mexico and in other parts of the world. Full details of the data available may also be viewed at https://datashare.ndph.ox.ac.uk/. Finngen release seven (r7) genetic association results, which were used in the current study are publicly available at https://r7.finngen.fi/.

Human research participants

Policy information about studies involving human research participants and Sex and Gender in Research.

Reporting on sex and gender	Sex is included as a covariate in the genetic association analysis. Sex is inferred from the genetic data and was confirmed by comparing with the self reported sex. We did not use gender information for any of the analysis.
Population characteristics	Provided in the supplementary table 2
Recruitment	Participant recruitment information for the respective cohorts is described in the methods section along with appropriate references.
Ethics oversight	All the study participants have provided informed consent and all the participating cohorts have received ethical approval from their respective institutional review board (IRB). The UK Biobank project has received ethical approval from the Northwest Centre for Research Ethics Committee (11/NW/0382). The work described here has been approved by the UKB (application no. 26041). The GHS project has received ethical approval from the Geisinger Health System Institutional Review Board under project no. 2006-025862. The MCPS study has received ethical approval from the Mexican Ministry of Health, the Mexican National Council for Science and Technology, and the University of Oxford. The BioMe biobank has received ethical approval from the IRB at the Icahn School of Medicine at Mount Sinai.

Note that full information on the approval of the study protocol must also be provided in the manuscript.

Field-specific reporting

Please select the one below that is the best fit for your research. If you are not sure, read the appropriate sections before making your selection.

☒ Life sciences ☐ Behavioural & social sciences ☐ Ecological, evolutionary & environmental sciences

For a reference copy of the document with all sections, see nature.com/documents/nr-reporting-summary-flat.pdf

Life sciences study design

All studies must disclose on these points even when the disclosure is negative.

Sample size	Sample size was not calculated prior to study. All samples available after quality control were included for analysis.
Data exclusions	Certain samples and genetic variants were excluded as part of the standard quality control pipeline applicable to any genetic association study. Details can be found in the methods and the cited references.
Replication	We did not have a separate replication cohort internally. We pooled genetic data from all our internal cohorts (UKB, GHS, MCPS and SINAI) to perform a meta-analysis. We identified three significant genes (ASXL1, DNMT3A and CHRNB2) for which we looked for consistency in the effect size directions and evidence for statistical significance (P<0.05) in the individual cohorts. The meta-analysis and individual cohort results of all three genes are reported in the manuscript. For all three genes, we observed a consistent direction of effect in at least three cohorts. and both a consistent direction of effect and statistical significance in at least two cohorts (Fig. 3). In addition, we replicated the protective association of a rare missense variant (Arg460Gly) with smoking-related phenotypes (substance use disorder and COPD) using the publicly available genetic association results from Finngen (release 7).
Randomization	Randomization is not applicable or possible in this study as it is a genetic association study based on hundreds of thousands of humans whose phenotypic information were collected retrospectively from the Electronic Health Records or health questionnaires responses of the participants or patients.
Blinding	Blinding is not required in this study as the phenotyping, genotyping and statistical analyses are completely independent processes and each happened without any prior knowledge of the others.

Reporting for specific materials, systems and methods

We require information from authors about some types of materials, experimental systems and methods used in many studies. Here, indicate whether each material, system or method listed is relevant to your study. If you are not sure if a list item applies to your research, read the appropriate section before selecting a response.

Materials & experimental systems

n/a	Involved in the study
☒	☐ Antibodies
☒	☐ Eukaryotic cell lines
☒	☐ Palaeontology and archaeology
☒	☐ Animals and other organisms
☒	☐ Clinical data
☒	☐ Dual use research of concern

Methods

n/a	Involved in the study
☒	☐ ChIP-seq
☒	☐ Flow cytometry
☒	☐ MRI-based neuroimaging
